# Mortality among hospitalized COVID-19 patients during surges of SARS-CoV-2 alpha (B.1.1.7) and delta (B.1.617.2) variants

**DOI:** 10.1038/s41598-022-23312-8

**Published:** 2022-11-07

**Authors:** Alireza Zali, Mahmood Khodadoost, Saeid Gholamzadeh, Shahriar Janbazi, Hassan Piri, Nazanin Taraghikhah, Khatereh Hannani, Mehdi Azizmohammad Looha, Gohar Mohammadi

**Affiliations:** 1 Functional Neurosurgery Research Center, Shohada Tajrish Comprehensive Neurosurgical Center of Excellence, Shahid Beheshti University of Medical Sciences, Tehran, Iran; 2 School of Traditional Medicine, Traditional Medicine and Materia Medica Research Center, Shahid Beheshti University of Medical Sciences, Tehran, Iran; 3 Administration and Resources Development affairs, Shahid Beheshti University of Medical Sciences, Tehran, Iran; 4 Legal Medicine Research Center, Legal Medicine Organization, Tehran, Iran; 5 Department of Health and Medical Medicine, Faculty of Medicine, Shahid Beheshti University of Medical Sciences, Tehran, Iran; 6 School of Traditional Medicine, Shahid Beheshti University of Medical Sciences, Tehran, Iran; 7 Gastroenterology and Liver Diseases Research Center, Research Institute for Gastroenterology and Liver Diseases, Shahid Beheshti University of Medical Sciences, Tehran, Iran; 8 Statistics and Information Technology Management, Shahid Beheshti University of Medical Sciences, Tehran, Iran; 9 Basic and Molecular Epidemiology of Gastrointestinal Disorders Research Center, Research Institute for Gastroenterology and Liver Diseases, Shahid Beheshti University of Medical Sciences, Tehran, Iran

**Keywords:** Diseases, Health care, Medical research, Risk factors, Signs and symptoms

## Abstract

The aim of this study was to evaluate the death proportion and death risk of COVID-19 hospitalized patients over time and in different surges of COVID-19. This multi-center observational study was conducted from March 21, 2021 to October 3, 2021 which included the alpha and delta SARS-CoV-2 surges occurred in April and August in Tehran, respectively. The risk of COVID-19 death was compared in different months of admission. A total of 270,624 patients with COVID-19, of whom 6.9% died, were admitted to hospitals in Tehran province. Compared to patients admitted in March, a higher risk of COVID-19 death was observed among patients admitted to the hospital in July (HR 1.28; 95% CI 1.17, 1.40), August (HR 1.40; 95% CI 1.28, 1.52), September (HR 1.37; 95% CI 1.25, 1.50) and October (HR 4.63; 95% CI 2.77, 7.74). The ICU death proportion was 36.8% (95% CI: 35.5, 38.1) in alpha surge and increased significantly to 39.8 (95% CI 38.6, 41.1) in delta surge. The risk of COVID-19 death was significantly higher in delta surge compared to alpha surge (HR 1.22; 95% CI 1.17, 1.27). Delta surge was associated with a higher risk of death compared to alpha surge. High number of hospitalizations, a shortage of hospital beds, ICU spaces and medical supplies, poor nutritional status of hospitalized patients, and lack of the intensivist physicians or specialized nurses in the ICU were factors that contributed to the high mortality rate in the delta surge in Iran.

## Introduction

Severe acute respiratory syndrome coronavirus 2 (SARS‐CoV‐2) has been a major health issue causing thousands of deaths daily worldwide since December 2019^1^. Iran has been among the most prone countries to this novel virus, which was first documented in Qom province on 19th February 2019^2^. As of 20th November 2021, more than 5,000,000 deaths globally had been attributed to Coronavirus disease 2019 (COVID-19), with over 129,000 deaths in Iran^3^.

Clinical experience has revealed variation in the trajectory of COVID-19, ranging from asymptomatic cases to those with mild, moderate, severe conditions, and even death^4,5^. Numerous factors have been identified as predictors of severe outcomes and death, such as increasing age, male gender, and comorbidities such as hypertension, diabetes, cardiac disease, chronic kidney disease, and liver disease^6^.

Over time, new variants of SARS-CoV-2 have emerged worldwide. WHO has approved three criteria to define variants: increased transmissibility, increase in virulence or change in clinical manifestations, and decrease in the efficacy of public health services, vaccines, and therapeutics^7^. In this study, alpha (B.1.1.7) and delta (B.1.617.2) variants were investigated. Alpha (B.1.1.7) variant was first detected in England in November, 2020, and transmitted rapidly to 154 countries, such as Iran^8^. Delta (B.1.617.2), the second variant, was first reported in India, in December 2020^9^. Several studies revealed that the delta variant was more transmissible with increased risk of hospitalization, intensive care unit (ICU) stay, and death in comparison to the alpha variant^10,11^.

The trend of mortality during the pandemic around the world is varied and difficult to describe due to the different stages of COVID-19 waves, periods of time, diverse underlying medical conditions of patients, and hospital load^12^. Moreover, mortality trend followed ICU-mortality trend, which was associated with the load of cases admitted to the ICU during the pandemic. In a study from Spain, the ICU mortality rate increased by 30% during the second/third wave when bed capacity peaked over 25%^1^. The trend of mortality over time should also be evaluated between waves, since the rapid influx of patients could adversely affect their outcomes.

Assessing COVID-19 mortality trends and identifying its risk factors can be a solution to guide healthcare providers in their decision-making and make the most of their abilities and facilities to immediately detect and save at-risk patients. To further expand this knowledge, this multicenter study mainly aimed to assess the death proportion and death risk of COVID-19 hospitalized cases over time in Tehran.

## Material and methods

### Study design and setting

This is a multi-center observational study involving hospitalized COVID-19 patients in Tehran province from March 21, 2021 to October 3, 2021. All methods were performed in accordance with the relevant guidelines and regulations.

### Study participants

The study was conducted using the registry database of Coronavirus Control Operations Headquarter in the province of Tehran. According to the latest national census conducted in 2016, there were 13,267,637 people living in the province. Of these, 8,693,706 lived in urban areas. This region is a major COVID-19 epicenter and the most populous in the country^13^.

The Iranian nation's central registry for novel coronavirus diseases was established in March 2020. All suspected, probable, and confirmed cases of COVID-19 were prospectively recorded on the national registry of COVID-19 database following WHO definition guidelines^14^. This multi- center study included all cases of COVID-19 who referred to more than 170 COVID-19 designated healthcare facilities in the province of Tehran between March 2021 and October 2021.

### Study variables and outcome

The outcome of interest was mortality among hospitalized patients with COVID-19. In this study, all days of hospitalization were considered. Patients who were discharged or lost to follow up were regarded as censored cases. The survival time was the period between the admission date and the death or discharge date.

Demographic variables (age, sex, smoking, and nationality), signs and symptoms (vomit, diarrhea, anorexia, paralysis, fever, seizure, muscular pain, chest pain, abdominal pain, respiratory distress, nausea, headache, cough, vertigo, skin lesion, loss of taste and anosmia), comorbidities (heart disease, HIV, asthma, neurological disease, hypertension, hematologic disease, liver disease, kidney disease, diabetes, cancer, immunodeficiency, and other chronic disease), drug abuse, CT results, residence county, and wards were included in this study as the variables.

### Statistical analyses

#### Descriptive analysis

Descriptive statistics were presented using frequency (percentage) and median (interquartile range [IQR]) for categorical and continuous variables, respectively, by month of admission, ward and death status. The 95% confidence interval (CI) for proportions was calculated using Newcombe methods^15^. In order to measure the association of outcome with categorical and continuous variables, Cramer's V and Eta were used, respectively. Cramer’s V and Eta range from 0 to 1, where 0 indicates no relationship and 1 indicates perfect association.

Density plot was used to illustrate the new cases of COVID-19 over the study period by death status, age category and ward.

#### Survival and mortality analysis

The adjusted effects of variables such as admission month, sex, age category, number of comorbidities, and nationality on the time to death from COVID-19 were assessed using the Cox proportional method and presented as forest plots. The supplementary file contains estimates of hazard ratio (HR) and 95% CIs. The crude death proportion was illustrated using line plots by sex, age, number of comorbidities, nationality and ward over the study period. In addition, the death proportion was presented for each county of Tehran province.

#### Subgroup survival and mortality analysis

Using a subgroup Cox proportional analysis, the effect of month of admission on the risk of COVID-19 death was evaluated after adjusting for gender, age, number of comorbidities, and nationality in different subgroups of ICU patients and non-ICU patients. In addition, the unadjusted impact of admission month was assessed in different subgroups of age category, sex, number of comorbidities, and nationality. In the supplementary file, all HR estimates, HR-related confidence intervals, and death-related confidence intervals are included.

### Ethics approval and consent to participate

Shahid Beheshti University of Medical Sciences Ethics Committee approved the study with a waiver of informed consent (Reference number: IR.SBMU.RETECH.REC.1400.473). All data were de-identified prior to analysis.

## Results

### Study population

**Figure 1:**
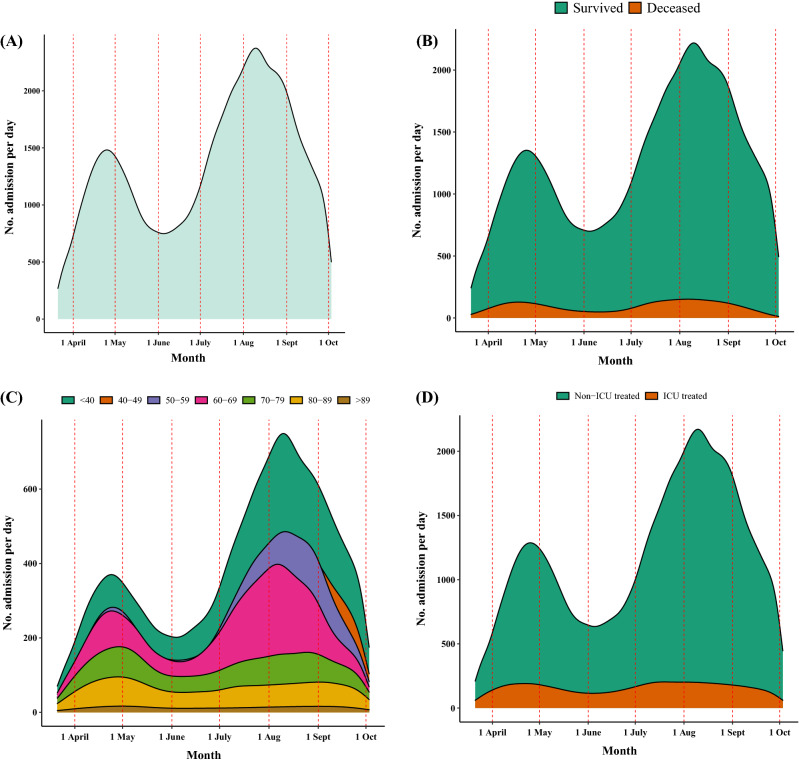
Density plot of patients’ frequency per Month during the study period. (**A**) Hospital admissions per month, (**B**) by death status, (**C**) by age group, and (**D**) by wards of hospital.

A total of 270,624 patients with COVID-19 were admitted to hospitals in Tehran province between 21st March, 2021 and 3rd October, 2021. The median age of COVID-19 patients was 50 (IQR: 37, 64) years and 50.2% of patients were male. As shown in Fig. 1A, two peaks occurred in the number of patients admitted to hospitals at the end of April and in the middle of August. These peaks were related respectively to the alpha and delta variants. Figure 1B showed that the difference in death frequencies between the two peaks was less than the difference in admission frequencies. According to Fig. 1C, the youngest category of patients was more frequent during the second peak. As shown in Fig. 1D, the number of patients treated in ICUs and those not treated in ICUs did not differ between the first and second peaks.

Patients’ characteristics were summarized by month of admission, as shown in Table 1 (see Supplementary Table 1, Additional File 1). Accordingly, the proportion of men was 51.9 (95% CI 50.6, 53.2) in March, declining to 47.3 (45.2, 49.5) by October. In March, the median age was 55 (IQR: 39, 68), and in October, it fell to 48 (36, 65). In April (first peak), the proportion of patients aged younger than 40, 40–49, and 50–59 was 25.5 (95% CI 25.1, 26.0), 16.5 (95% CI 16.1, 16.8), and 19.1 (95% CI 18.7, 19.5) respectively, which was increased to 31.4 (95% CI 32.0, 32.9), 20.0 (95% CI 19.7, 20.3), and 21.0 (95% CI 20.7, 21.3) in August (second peak). The trend of admission proportion was reversed for those aged 60 and older; lower patients’ proportions were noted in August as compared to April.

**Table 1:** Characteristics of patients at baseline, stratified by month of admission.

	Total	March	April	May	June	July	August	September	October
Total (No.)	270,624	5743	37,599	32,483	25,405	53,128	70,187	44,007	2072
**Sex (No.,%)**									
Women	134,693 (49.8)	2764 (48.1)	18,140 (48.2)	15,801 (48.6)	12,390 (48.8)	26,106 (49.1)	35,625 (50.8)	22,776 (51.8)	1091 (52.7)
Men	135,931 (50.2)	2979 (51.9)	19,459 (51.8)	16,682 (51.4)	13,015 (51.2)	27,022 (50.9)	34,562 (49.2)	21,231 (48.2)	981 (47.3)
Median age (IQR)	50 (37, 64)	55 (39, 68)	54 (39, 67)	54 (39, 67)	53 (38, 66)	50 (37, 62)	49 (37, 61)	48 (36, 62)	48 (36, 65)
**Age (No. %)**									
< 40	79,161 (29.3)	1488 (25.9)	9604 (25.5)	8178 (25.2)	7014 (27.6)	15,887 (29.9)	22,027 (31.4)	14,276 (32.4)	687 (33.2)
40–49	50,495 (18.7)	871 (15.2)	6192 (16.5)	5321 (16.4)	4200 (16.5)	10,272 (19.3)	14,020 (20)	9227 (21)	392 (18.9)
50–59	52,646 (19.5)	988 (17.2)	7164 (19.1)	5994 (18.5)	4685 (18.4)	10,767 (20.3)	14,727 (21)	7982 (18.1)	339 (16.4)
60–69	45,935 (17.0)	1080 (18.8)	7034 (18.7)	5999 (18.5)	4608 (18.1)	9487 (17.9)	11,603 (16.5)	5844 (13.3)	280 (13.5)
70–79	25,783 (9.5)	778 (13.5)	4591 (12.2)	4202 (12.9)	2969 (11.7)	4176 (7.9)	4946 (7)	3895 (8.9)	226 (10.9)
80–89	13,966 (5.2)	452 (7.9)	2565 (6.8)	2353 (7.2)	1601 (6.3)	2166 (4.1)	2385 (3.4)	2317 (5.3)	127 (6.1)
> 89	2638 (1.0)	1488 (25.9)	9604 (25.5)	8178 (25.2)	7014 (27.6)	15,887 (29.9)	22,027 (31.4)	14,276 (32.4)	687 (33.2)
**Nationality (No.,%)**									
Iranian	262,512 (97.0)	189 (3.3)	995 (2.6)	791 (2.4)	946 (3.7)	1777 (3.3)	1920 (2.7)	1425 (3.2)	69 (3.3)
Non-Iranian	8112 (3.0)	5554 (96.7)	36,604 (97.4)	31,692 (97.6)	24,459 (96.3)	51,351 (96.7)	68,267 (97.3)	42,582 (96.8)	2003 (96.7)
**Sign and symptoms (No.%)**									
Vomit	9503 (3.5)	296 (5.2)	1553 (4.1)	1420 (4.4)	1150 (4.5)	1626 (3.1)	1933 (2.8)	1464 (3.3)	61 (2.9)
Diarrhea	8031 (3.0)	264 (4.6)	1471 (3.9)	1208 (3.7)	1006 (4.0)	1387 (2.6)	1637 (2.3)	1025 (2.3)	33 (1.6)
Anorexia	26,038 (9.6)	588 (10.2)	3565 (9.5)	3114 (9.6)	2469 (9.7)	4765 (9.0)	7172 (10.2)	4172 (9.5)	193 (9.3)
Paralysis	276 (0.1)	10 (0.2)	37 (0.1)	47 (0.1)	29 (0.1)	60 (0.1)	55 (0.1)	38 (0.1)	(0.0)
Fever	104,056 (38.5)	2194 (38.2)	13,530 (36.0)	11,428 (35.2)	9914 (39.0)	21,286 (40.1)	27,865 (39.7)	17,124 (38.9)	715 (34.5)
Seizure	760 (0.3)	33 (0.6)	107 (0.3)	105 (0.3)	99 (0.4)	136 (0.3)	155 (0.2)	112 (0.3)	13 (0.6)
Muscular pain	102,184 (37.8)	2147 (37.4)	13,616 (36.2)	11,626 (35.8)	8788 (34.6)	20,097 (37.8)	27,422 (39.1)	17,679 (40.2)	809 (39.0)
Chest pain	7311 (2.7)	198 (3.4)	1412 (3.8)	1015 (3.1)	715 (2.8)	1169 (2.2)	1682 (2.4)	1079 (2.5)	41 (2.0)
Abdominal pain	6559 (2.4)	143 (2.5)	1156 (3.1)	809 (2.5)	637 (2.5)	1165 (2.2)	1700 (2.4)	919 (2.1)	30 (1.4)
Dyspnea	111,113 (41.1)	2678 (46.6)	17,898 (47.6)	14,085 (43.4)	9908 (39.0)	22,007 (41.4)	27,312 (38.9)	16,394 (37.3)	831 (40.1)
Nausea	18,618 (6.9)	480 (8.4)	2630 (7.0)	2136 (6.6)	1819 (7.2)	3427 (6.5)	4991 (7.1)	2998 (6.8)	137 (6.6)
Headache	29,143 (10.8)	516 (9.0)	3825 (10.2)	3464 (10.7)	2460 (9.7)	5830 (11.0)	7839 (11.2)	5004 (11.4)	205 (9.9)
Cough	158,910 (58.7)	2999 (52.2)	20,365 (54.2)	17,764 (54.7)	13,597 (53.5)	31,314 (58.9)	44,401 (63.3)	27,273 (62.0)	1197 (57.8)
Vertigo	8962 (3.3)	185 (3.2)	1372 (3.6)	1177 (3.6)	809 (3.2)	1657 (3.1)	2262 (3.2)	1419 (3.2)	81 (3.9)
Skin lesion	249 (0.1)	6 (0.1)	30 (0.1)	29 (0.1)	21 (0.1)	47 (0.1)	65 (0.1)	48 (0.1)	3 (0.1)
Loss of taste	4841 (1.8)	94 (1.6)	488 (1.3)	409 (1.3)	428 (1.7)	982 (1.8)	1526 (2.2)	890 (2.0)	24 (1.2)
Anosmia	8335 (3.1)	120 (2.1)	753 (2.0)	1024 (3.2)	787 (3.1)	1752 (3.3)	2183 (3.1)	1677 (3.8)	39 (1.9)
**Comorbidities (No.%)**									
Heart disease	18,541 (6.9)	621 (10.8)	3289 (8.7)	3080 (9.5)	2353 (9.3)	3021 (5.7)	3599 (5.1)	2447 (5.6)	131 (6.3)
HIV	90 (0.0)	6 (0.1)	15 (0.0)	16 (0.0)	10 (0.0)	18 (0.0)	18 (0.0)	7 (0.0)	(0.0)
Asthma	1878 (0.7)	59 (1.0)	363 (1.0)	308 (0.9)	225 (0.9)	342 (0.6)	342 (0.5)	230 (0.5)	9 (0.4)
Neurological disease	1251 (0.5)	51 (0.9)	215 (0.6)	211 (0.6)	147 (0.6)	191 (0.4)	226 (0.3)	197 (0.4)	13 (0.6)
Hypertension	26,046 (9.6)	776 (13.5)	4602 (12.2)	4217 (13.0)	3068 (12.1)	4430 (8.3)	5346 (7.6)	3406 (7.7)	201 (9.7)
Hematologic disease	839 (0.3)	26 (0.5)	102 (0.3)	108 (0.3)	101 (0.4)	144 (0.3)	195 (0.3)	153 (0.3)	10 (0.5)
Liver disease	810 (0.3)	29 (0.5)	124 (0.3)	134 (0.4)	108 (0.4)	138 (0.3)	155 (0.2)	119 (0.3)	3 (0.1)
Kidney disease	2475 (0.9)	91 (1.6)	402 (1.1)	401 (1.2)	319 (1.3)	426 (0.8)	494 (0.7)	311 (0.7)	31 (1.5)
Diabetes	22,191 (8.2)	669 (11.6)	3830 (10.2)	3416 (10.5)	2532 (10.0)	3881 (7.3)	4647 (6.6)	3065 (7.0)	151 (7.3)
Other chronic diseases	10,271 (3.8)	315 (5.5)	1594 (4.2)	1629 (5.0)	1272 (5.0)	1780 (3.4)	2075 (3.0)	1519 (3.5)	87 (4.2)
Cancer	2349 (0.9)	70 (1.2)	419 (1.1)	412 (1.3)	313 (1.2)	367 (0.7)	390 (0.6)	360 (0.8)	18 (0.9)
Immunodeficiency	483 (0.2)	18 (0.3)	73 (0.2)	84 (0.3)	44 (0.2)	73 (0.1)	94 (0.1)	91 (0.2)	6 (0.3)
**No. of comorbidities (No.%)**									
0	212,378 (78.5)	3960 (69.0)	27,517 (73.2)	23,300 (71.7)	18,538 (73.0)	43,062 (81.1)	58,267 (83.0)	36,098 (82.0)	1636 (79.0)
1	36,041 (13.3)	1061 (18.5)	6244 (16.6)	5541 (17.1)	4119 (16.2)	6393 (12.0)	7577 (10.8)	4836 (11.0)	270 (13.0)
2	16,405 (6.1)	529 (9.2)	2870 (7.6)	2615 (8.1)	1992 (7.8)	2764 (5.2)	3211 (4.6)	2309 (5.2)	115 (5.6)
3	4932 (1.8)	160 (2.8)	837 (2.2)	879 (2.7)	652 (2.6)	759 (1.4)	964 (1.4)	637 (1.4)	44 (2.1)
≥ 4	868 (0.3)	33 (0.6)	131 (0.3)	148 (0.5)	104 (0.4)	150 (0.3)	168 (0.2)	127 (0.3)	7 (0.3)
**Pregnancy (No.%)**									
No	269,083 (99.4)	5690 (99.1)	37,334 (99.3)	32,295 (99.4)	25,246 (99.4)	52,857 (99.5)	69,840 (99.5)	43,759 (99.4)	2062 (99.5)
Yes	1541 (0.6)	53 (0.9)	265 (0.7)	188 (0.6)	159 (0.6)	271 (0.5)	347 (0.5)	248 (0.6)	10 (0.5)
**Smoking (No.%)**									
No	267,436 (98.8)	5637 (98.2)	37,094 (98.7)	32,051 (98.7)	25,009 (98.4)	52,500 (98.8)	69,541 (99.1)	43,552 (99.0)	2052 (99.0)
Yes	3188 (1.2)	106 (1.8)	505 (1.3)	432 (1.3)	396 (1.6)	628 (1.2)	646 (0.9)	455 (1.0)	20 (1.0)
**Drug abuse (No.%)**									
No	269,342 (99.5)	5692 (99.1)	37,389 (99.4)	32,293 (99.4)	25,203 (99.2)	52,916 (99.6)	69,958 (99.7)	43,828 (99.6)	2063 (99.6)
Yes	1282 (0.5)	51 (0.9)	210 (0.6)	190 (0.6)	202 (0.8)	212 (0.4)	229 (0.3)	179 (0.4)	9 (0.4)
**CT Scan (No.%)**									
Negative	67,715 (25.0)	1638 (28.5)	9767 (26.0)	8468 (26.1)	7645 (30.1)	13,223 (24.9)	16,334 (23.3)	10,102 (23.0)	538 (26.0)
Positive	202,909 (75.0)	4105 (71.5)	27,832 (74.0)	24,015 (73.9)	17,760 (69.9)	39,905 (75.1)	53,853 (76.7)	33,905 (77.0)	1534 (74.0)
**Ward**									
Non-ICU treated	238,575 (88.2)	4480 (78.0)	32,095 (85.4)	28,018 (86.3)	21,572 (84.9)	47,057 (88.6)	64,135 (91.4)	39,372 (89.5)	1846 (89.1)
ICU treated	32,049 (11.8)	1263 (22.0)	5504 (14.6)	4465 (13.7)	3833 (15.1)	6071 (11.4)	6052 (8.6)	4635 (10.5)	226 (10.9)
**Death status**									
Survived	252,001 (93.1)	5174 (90.1)	34,004 (90.4)	30,040 (92.5)	23,883 (94.0)	49,262 (92.7)	65,701 (93.6)	41,880 (95.2)	2057 (99.3)
Deceased	18,623 (6.9)	569 (9.9)	3595 (9.6)	2443 (7.5)	1522 (6.0)	3866 (7.3)	4486 (6.4)	2127 (4.8)	15 (0.7)
Hospitalization days. median (IQR)	5 (2, 7)	5 (2, 7)	5 (2, 7)	5 (3, 7)	5 (2, 7)	5 (2, 7)	5 (2, 7)	4 (2, 6)	1 (1, 1)

*Abbreviation*: Intensive care unit (ICU), HIV (human immunodeficiency virus).

### Overall morality

18,623 (6.88%) patients with COVID-19 died during the study period. The death proportion by ward (non-ICU and ICU treated) and patient status (survived and deceased) is presented in Table 2 (see Supplementary Table 2, Additional File 1). The death proportion for patients with COVID-19 admitted to non-ICU wards was 3.2 (95% CI 3.2–3.3), while it was 34.0 (95% CI 33.5–34.5) for those admitted to ICUs. The strongest associations were observed between outcome (survived and deceased) and age (EF = 0.23), hospitalization days (EF = 0.21), respiratory distress (EF = 0.14), number of comorbidities (EF = 0.07), kidney disease (EF = 0.06), chest pain (EF = 0.05), hypertension (EF = 0.05), and diabetes (EF = 0.05).

**Table 2:** Patient characteristics stratified by Ward.

	Non-ICU treated		EF (*p* value)	ICU treated		EF (*p* value)
	Survived	Deceased		Survived	Deceased	
Total (No. %)	230,846 (96.8)	7729 (3.2)		21,155 (66.0)	10,894 (34.0)	
**Sex (No.,%)**			0.02 (< 0.001)			0.03 (< 0.001)
Women	116,610 (50.5)	3397 (44.0)		9920 (46.9)	4766 (43.7)	
Men	114,236 (49.5)	4332 (56.0)		11,235 (53.1)	6128 (56.3)	
**Age**			0.21 (< 0.001)			0.23 (< 0.001)
< 40	73,582 (31.9)	541 (7.0)		4314 (20.4)	724 (6.6)	
40–49	46,067 (20.0)	555 (7.2)		2901 (13.7)	972 (8.9)	
50–59	46,028 (19.9)	1167 (15.1)		3743 (17.7)	1708 (15.7)	
60–69	36,828 (16.0)	1901 (24.6)		4464 (21.1)	2742 (25.2)	
70–79	18,479 (8.0)	1666 (21.6)		3255 (15.4)	2383 (21.9)	
80–89	8517 (3.7)	1514 (19.6)		2057 (9.7)	1878 (17.2)	
> 89	1345 (0.6)	385 (5.0)		421 (2.0)	487 (4.5)	
**Nationality (No.,%)**			-0.1 (< 0.001)			0.00 (0.651)
Iranian	224,123 (97.1)	7417 (96.0)		20,451 (96.7)	10,521 (96.6)	
Non-Iranian	6723 (2.9)	312 (4.0)		704 (3.3)	373 (3.4)	
**Sign and Symptoms (No.%)**						
Vomit	8198 (3.6)	162 (2.1)	0.01 (< 0.001)	768 (3.6)	375 (3.4)	0.00 (0.390)
Diarrhea	7157 (3.1)	123 (1.6)	0.02 (< 0.001)	518 (2.4)	233 (2.1)	0.01 (0.082)
Anorexia	22,209 (9.6)	577 (7.5)	0.01 (< 0.001)	1951 (9.2)	1301 (11.9)	0.04 (< 0.001)
Paralysis	176 (0.1)	14 (0.2)	0.01 (0.001)	49 (0.2)	37 (0.3)	0.01 (0.077)
Fever	90,621 (39.3)	2710 (35.1)	0.02 (< 0.001)	7044 (33.3)	3681 (33.8)	0.00 (0.376)
Seizure	615 (0.3)	8 (0.1)	0.01 (0.006)	112 (0.5)	25 (0.2)	0.02 (< 0.001)
Muscular pain	89,161 (38.6)	2473 (32.0)	0.02 (< 0.001)	6828 (32.3)	3722 (34.2)	0.02 (< 0.001)
Chest pain	5316 (2.3)	163 (2.1)	0.00 (0.263)	1391 (6.6)	441 (4.0)	0.05 (< 0.001)
Abdominal pain	5764 (2.5)	138 (1.8)	0.01 (< 0.001)	425 (2.0)	232 (2.1)	0.00 (0.470)
Dyspnea	87,383 (37.9)	4825 (62.4)	0.09 (< 0.001)	11,444 (54.1)	7461 (68.5)	0.14 (< 0.001)
Nausea	16,278 (7.1)	295 (3.8)	0.02 (< 0.001)	1325 (6.3)	720 (6.6)	0.01 (0.230)
Headache	26,129 (11.3)	446 (5.8)	0.03 (< 0.001)	1641 (7.8)	927 (8.5)	0.01 (0.019)
Cough	139,509 (60.4)	3655 (47.3)	0.05 (< 0.001)	10,140 (47.9)	5606 (51.5)	0.03 (< 0.001)
Vertigo	7592 (3.3)	162 (2.1)	0.01 (< 0.001)	790 (3.7)	418 (3.8)	0.00 (0.648)
Skin lesion	206 (0.1)	7 (0.1)	0.00 (0.969)	23 (0.1)	13 (0.1)	0.00 (0.788)
Loss of taste	4220 (1.8)	42 (0.5)	0.02 (< 0.001)	414 (2.0)	165 (1.5)	0.02 (0.005)
Anosmia	7341 (3.2)	137 (1.8)	0.01 (< 0.001)	548 (2.6)	309 (2.8)	0.01 (0.196)
**Comorbidities (No.%)**						
Heart disease	12,346 (5.3)	1080 (14.0)	0.07 (< 0.001)	3221 (15.2)	1894 (17.4)	0.03 (< 0.001)
HIV	61 (0.0)	5 (0.1)	0.00 (0.047)	13 (0.1)	11 (0.1)	0.01 (0.221)
Asthma	1476 (0.6)	65 (0.8)	0.00 (0.030)	224 (1.1)	113 (1.0)	0.00 (0.858)
Neurological disease	868 (0.4)	58 (0.8)	0.01 (< 0.001)	184 (0.9)	141 (1.3)	0.02 (< 0.001)
Hypertension	18,640 (8.1)	1179 (15.3)	0.05 (< 0.001)	3828 (18.1)	2399 (22.0)	0.05 (< 0.001)
Hematologic diseases	635 (0.3)	37 (0.5)	0.01 (< 0.001)	111 (0.5)	56 (0.5)	0.00 (0.900)
Liver disease	592 (0.3)	53 (0.7)	0.01 (< 0.001)	96 (0.5)	69 (0.6)	0.01 (0.033)
Kidney disease	1589 (0.7)	181 (2.3)	0.03 (< 0.001)	333 (1.6)	372 (3.4)	0.06 (< 0.001)
Diabetes	16,049 (7.0)	1189 (15.4)	0.06 (< 0.001)	3022 (14.3)	1931 (17.7)	0.05 (< 0.001)
Other chronic diseases	8363 (3.6)	287 (3.7)	0.00 (0.675)	978 (4.6)	643 (5.9)	0.03 (< 0.001)
Cancer	1541 (0.7)	166 (2.1)	0.03 (< 0.001)	343 (1.6)	299 (2.7)	0.04 (< 0.001)
Immunodeficiency	367 (0.2)	15 (0.2)	0.00 (0.448)	64 (0.3)	37 (0.3)	0.00 (0.575)
**No. of comorbidities (No.%)**			0.08 (< 0.001)			0.07 (< 0.001)
0	187,834 (81.4)	5094 (65.9)		13,296 (62.9)	187,834 (81.4)	
1	27,789 (12.0)	1379 (17.8)		4448 (21.0)	27,789 (12.0)	
2	11,497 (5.0)	905 (11.7)		2420 (11.4)	11,497 (5.0)	
3	3211 (1.4)	287 (3.7)		854 (4.0)	3211 (1.4)	
≥ 4	515 (0.2)	64 (0.8)		137 (0.6)	515 (0.2)	
**Pregnancy (No.%)**			0.01 (< 0.001)			0.04 (< 0.001)
No	229,447 (99.4)	7723 (99.9)		21,027 (99.4)	10,886 (99.9)	
Yes	1399 (0.6)	6 (0.1)		128 (0.6)	8 (0.1)	
**Smoking (No.%)**			0.00 (0.459)			0.04 (< 0.001)
No	228,591 (99.0)	7660 (99.1)		20,492 (96.9)	10,693 (98.2)	
Yes	2255 (1.0)	69 (0.9)		663 (3.1)	201 (1.8)	
**Drug abuse (No.%)**			0.01 (< 0.001)			0.02 (< 0.001)
No	230,119 (99.7)	7674 (99.3)		20,787 (98.3)	10,762 (98.8)	
Yes	727 (0.3)	55 (0.7)		368 (1.7)	132 (1.2)	
**CT Scan (No.%)**			0.02 (< 0.001)			0.11 (< 0.001)
Negative	58,148 (25.2)	1608 (20.8)		5979 (28.3)	1980 (18.2)	
Positive	172,698 (74.8)	6121 (79.2)		15,176 (71.7)	8914 (81.8)	
Hospitalization days. median (IQR)	4 (2, 6)	4 (2, 9)	0.13 (< 0.001)	6 (4, 9)	7 (3,12)	0.21 (< 0.001)

Note: The strength of relationship between categorical variable and outcome (survived and deceased) was presented using Cramer's V. The measure of association between continuous variable and outcome (survived and deceased) was showed using Eta method.

Abbreviation: Effect size (EF), intensive care unit (ICU).

**Figure 2:**
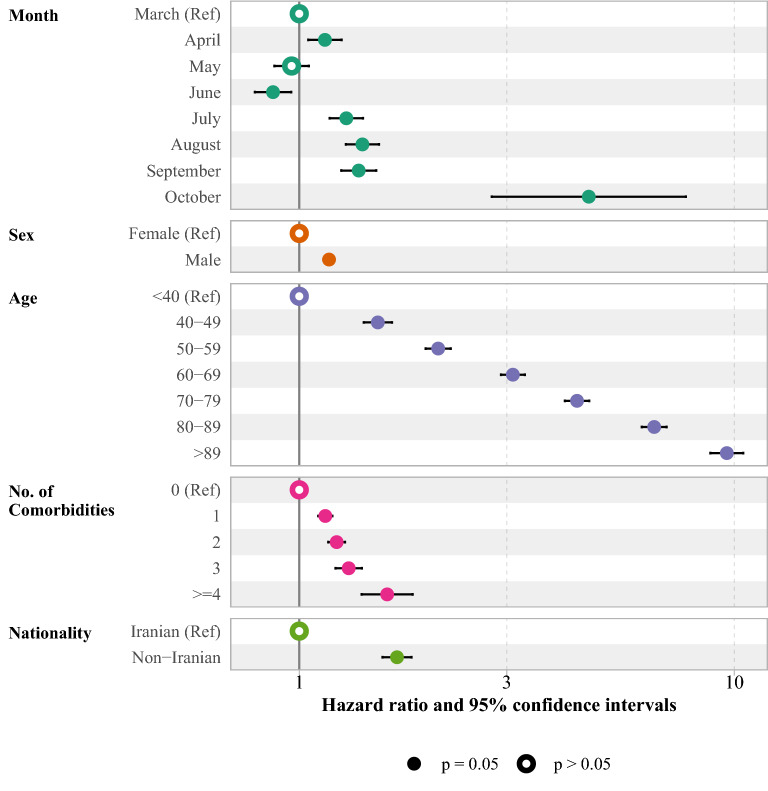
The forest plot of death hazard ratio among hospitalized patients of COVID-19. The Cox proportional model was adjusted for month of admission, sex, age, no. of comorbidities and nationality.

A multiple Cox regression was used to evaluate the adjusted risk of COVID-19 death among hospitalized patients, as shown in Fig. 2 (see Supplementary Table 3, Additional File 1). Compared to patients admitted in March, those admitted in June had lower risk of COVID-19 death (HR 0.87; 95% CI 0.79, 0.96). However, a higher risk of COVID-19 death was observed among patients admitted to the hospital in July (HR 1.28; 95% CI 1.17, 1.40), August (HR 1.40; 95% CI 1.28, 1.52), September (HR 1.37; 95% CI 1.25, 1.50) and October (HR 4.63; 95% CI 2.77, 7.74). The hazard of death for men was 17% higher than for women (HR 1.17; 95% CI 1.14, 1.21). The risk of COVID-19 death increased by age and the highest risk of death observed in those older than 89 years of age (HR 9.61; 95% CI 8.81, 10.49). COVID-19 death risk was associated with higher number of comorbidities, and those with more than three comorbidities were at higher risk (HR 1.59; 95% CI 1.39, 1.82).

Figure 6 shows that the highest death proportions were observed in counties including Malard (10.88%), Robat-Karim (8.81%) and Eslamshahr (8.48%). Among ICU-treated, the highest death proportions occurred in Pishva (52.03%), Varamin (46.64%), and Pakdasht (43.26%) (see Supplementary Fig. 2 and, Additional File 1).

### Trend of mortality over study period

**Figure 3:**
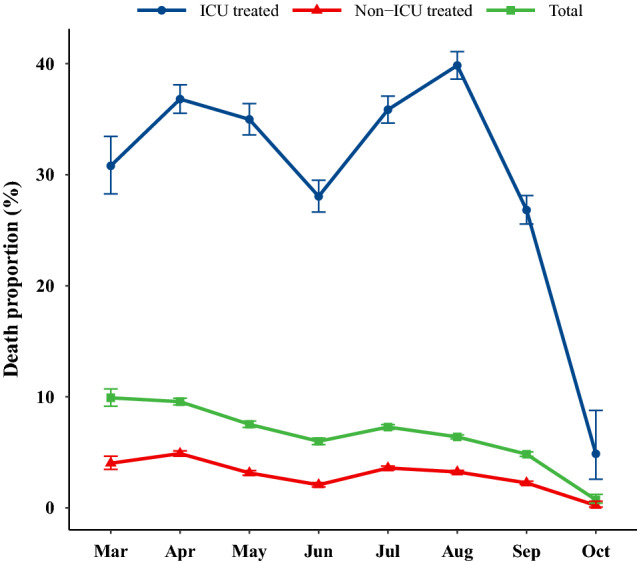
The trend of unadjusted death proportion due to COVID-19 among hospitalized patients per month. Error bars are the 95% confidence interval for death proportions.

Figure 3 (see Supplementary Table 4, Additional File 1) shows the trend of mortality for total patients, ICU and non-ICU treated by month of admission. Death proportion among ICU-treated increased from March (30.8; 95% CI 28.3, 33.4) to April (36.8; 95% CI 35.5, 38.1), remained constant until May (35.0; 95% CI 33.6, 36.4), and declined in January (28.0.8; 95% CI 26.6, 29.5) during the first peak of COVID-19. On the second peak, the death proportion increased in July (35.9, 95% CI 34.7, 37.1), then rose more to the top in August (39.8, 95% CI 38.6, 41.1), then declined in September (26.8, 95% CI 34.7, 37.1) and October (4.9, 95% CI 2.6, 8.8). Among non-ICU patients, however, mortality peaked in April (4.9, 95% CI 4.7, 5.1) and July (3.2, 95% CI 3.4, 3.8), respectively. Furthermore, the proportion of deaths was highest during the first peak among non-ICU patients.

**Figure 4:**
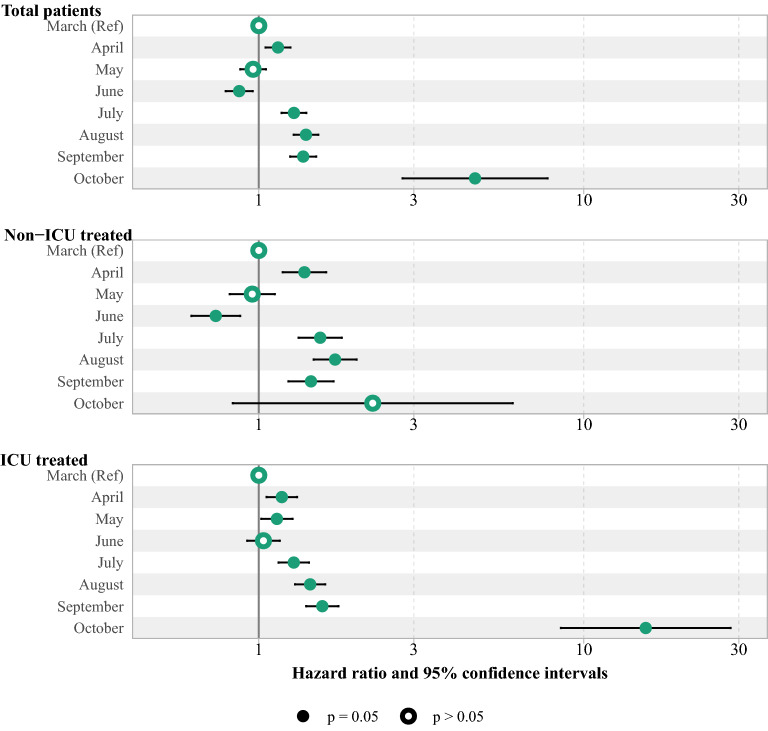
The adjusted HR and 95% CI of death among hospitalized COVID-19 patients by wards. The Cox proportional model was adjusted by variables including sex, age, No. of comorbidities and nationality.

The log-rank test shows the significant difference in survival rate between months. Accordingly, the survival rate in August was significantly lower than in April (see Supplementary Table 5 and Supplementary Fig. 1, Additional File 1). In addition, the risk of COVID-19 death was increased from 1.28 (95% CI 1.15, 1.43) in July to 15.51 (95% CI 8.50, 28.31) in August during the second peak among those admitted to the ICU. However, the highest risk of death among non-ICU patients was 1.72 (95% CI 1.47, 2.00) in August, as shown in Fig. 4 (see Supplementary Table 6, Additional File 1).

**Figure 5:**
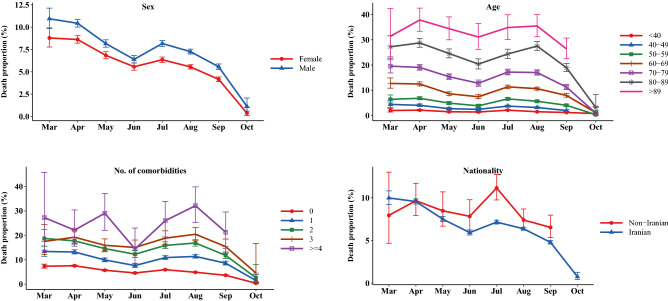
The death proportion and 95% confidence interval due to COVID-19 among hospitalized patients by sex, age, no. of comorbidities, and nationality.

According to Fig. 5, the death proportion was illustrated over time by sex, age group, number of comorbidities and nationality. Accordingly, an overall downward trend was observed with varying magnitudes over study time.

**Figure 6:**
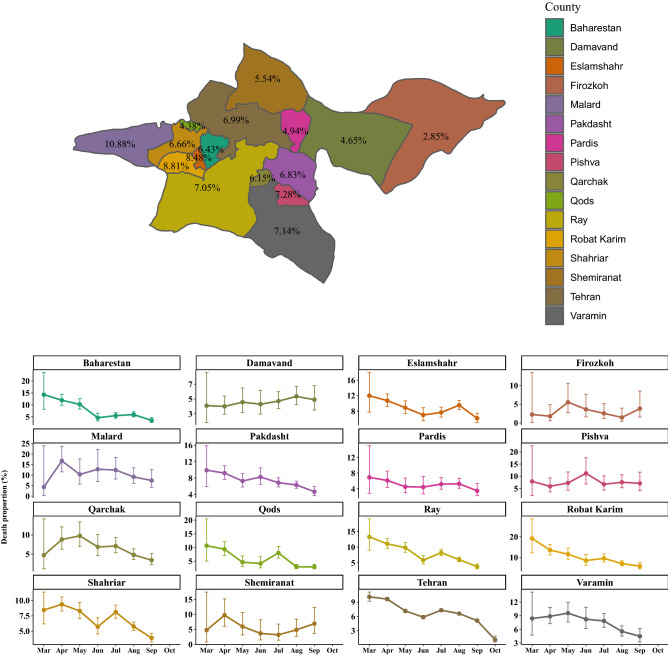
The overall death proportion (%) due to COVID-19 by Tehran County (upper panel). The monthly death proportion due to COVID-19 with 95% confidence interval by Tehran County (lower panel).

Nonetheless, the trend of death proportions varied from county to county in Tehran province, as shown in Fig. 6. Furthermore, the death proportion among ICU patients had risen in most counties until August, but declined for patients not receiving ICU treatment (see Supplementary Fig. 2 and 3, Additional File 1).

**Figure 7:**
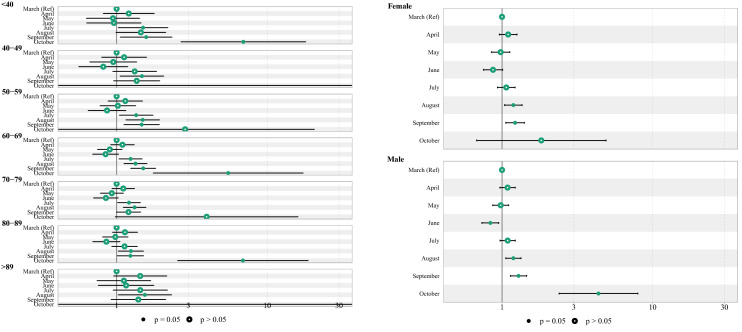

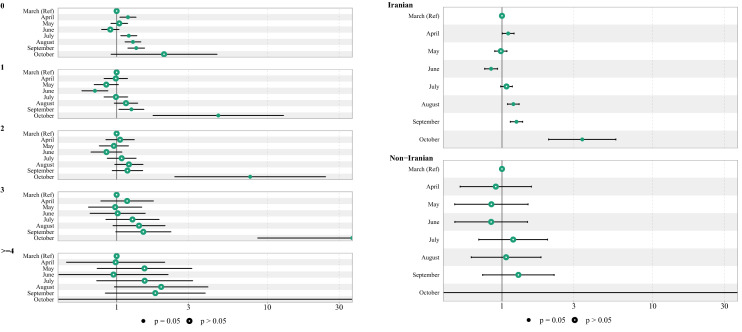
The subgroup HR and 95% CI of death among hospitalized COVID-19 patients by different group of age, sex, No. of comorbidities and nationality.

In the next step, the subgroup analysis was presented in Fig. 7. Accordingly, the month of admission had a different impact on mortality in various age groups, number of comorbidities and nationality, but there wasn't any difference in HR between males and females based on the month of admission.

## Discussion

In the current study, two COVID-19 peaks of hospitalization were identified between March and October 2021, which were often associated with alpha and delta variants, respectively. The first peak of hospitalizations was in April, followed by a second peak in August. The findings of this study were reported either as overall and by month of admission to the hospital. In the second peak, the proportion of patients under 60 years of age was significantly higher than in the first peak, while the proportion of patients aged 60 and older was significantly lower. Additionally, patients in the second peak were more likely to be female. The highest proportion of deaths was observed in the second and first peak of hospitalization, respectively. There was a ten-fold increase in mortality among ICU patients compared with those who were not. Although a downward trend was observed in the death proportion of patients in the ICU during the study period, it peaked in April and August. The study found decreasing mortality trends based on gender and age. Mortality was higher in patients with comorbidities greater than 3 during the second peak than the first, but the overall trend of mortality declined by comorbidities during the study period. As compared with March, the adjusted risk of COVID-19 death was higher among patients admitted to hospital in April, July, August, September, and October, however, among ICU patients, it was higher in all months except for June. The survival rate in the second peak was significantly lower than in the first peak. Overall, the highest death proportion occurred in Malard, Robat Karim, and Eslamshahr counties. Among ICU patients with COVID-19, Pishva, Varamin, and Pakdasht had the highest death proportion.

The variants of COVID-19 including alpha (B.1.1.7) and delta (B.1.617.2) were first detected in Iran in February and June, respectively^16,17^. Therefore, this study has found that the first and second peak of COVID-19 were associated with alpha and delta variants, respectively. In the current study, although the number of patients aged 59 years and younger admitted to the hospital were increased in the second peak than in the first peak, the death proportion decreased. In addition, the death proportion among patients infected with the delta variant of COVID-19 was lower than that of patients infected with the alpha variant. However, the second peak was associated with higher risk of COVID-19 death. These results were consistent with other studies which provided the most comprehensive assessment of hospitalization and death risk for both alpha and delta variants. Accordingly, in a study conducted by Twohig et al. on the 43,338 COVID-19-positive patients (8,682 with the delta variant, 34,656 with the alpha variant), higher proportion of hospital admission was founded for patients with COVID-19 infected with the delta variant compared with the alpha variant^18^. In another comprehensive study that compared COVID-19 patients in the pre-delta and delta surges, Bast et al. found that patients in the delta surge were more likely to be younger and female. Furthermore, delta surge was associated with a two-fold increase in death risk compared to pre-delta surge^11^. There are some issues that are debatable. The death proportion decreased from the first (alpha variant) to the second peak (delta variant) in this study, while the risk of COVID-19 death increased. The reason for reduction in death proportion might be that the number of cases admitted to the second peak increased rapidly, while deaths increased much more slowly. In contrast, it appears that the time to death was shorter for patients with the delta COVID-19 variant than for those with the alpha variant. Another issue was the start of public Coronavirus vaccination in Iran in May. In August, most people over the age of 60 had been vaccinated, and there was no significant increase in the death proportion in this age group. Despite the fact that most people in the younger age group were not yet vaccinated, the death proportion also decreased in this group. However, the number of admissions to the hospital in the second peak was reduced only for patients aged 60 years and older. Due to this, it is difficult to explain the effect of vaccination on reducing the hospitalization and death proportion in the second peak.

Our study found a higher death proportion and risk of COVID-19 death among ICU patients during the delta surge than the alpha surge. However, the mean age, mean number of comorbidities, and percent of men were lower in delta surge compared to the alpha surge. It may be due to the fact that the delta variant had a higher severity and fatality rate. According to Twohig et al., the delta variant is more likely to cause severe disease than the previously dominant alpha variant^18^. In addition, Mlcochova et al. found that delta variants showed higher replication efficiency in airway organoids and human epithelial systems than alpha variants. Therefore, delta variants were more transmissible^19^. It should also be taken into consideration that the second peak in Iran occurred only a few days after the first peak. This may result in fatigue, burnout, and frustration for the medical worker. In a study by Mollica et al., the importance of problems caused by mental and physical fatigue of health care providers was emphasized. Accordingly, many of these employees were willing to retire or leave their jobs^20^. Hu et al. found that frontline nurses suffered from a variety of mental health issues, including burnout and fear. In addition, a high rate of anxiety, depression, and skin lesions was found among these nurses as well^21^. According to the increased demand for specialists and nurses in ICU ward, all physicians and nurses were forced to work in ICU ward without any specialized training. This issue may be more difficult for health care providers to manage crises without regular and intensive training^22^. In this situation, the possibility of medical errors by physicians and nurses increases, which may lead to an increase in the mortality rate of patients in the ICU.

The current study revealed that there was a significant difference in death proportion among ICU patients in different counties of Tehran. The death proportion of patients in the ICU could be affected by a variety of factors, including nutritional status of the patient, the skill of the intensivist physician, the availability of qualified and experienced nurses, and the availability of modern medical equipment and laboratories. During the delta surge in Tehran, the number of available beds was insufficient to meet the patients' demands, and no new hospitals were built to increase bed supply. ICU bed shortages were more common in some counties. Therefore, patients were in a worse condition when admitted to the ICU after waiting for several hours to several days, which led to more deaths. Research by Xie et al. showed that mortality decreased as medical resources were integrated and developed. Moreover, a reduction in mortality rates was attributed to an increase in the number of medical staff^23^. Accordingly, one major factor contributing to the higher mortality rate in some counties is the fact that when the delta variant surged in Tehran, hospitals were overwhelmed, causing beds and ICU spaces to be rationed for patients who needed medical treatment. This situation would cause even healthcare workers to make challenging decisions about screening patients wisely to select who would be admitted and who would receive outpatient care. Furthermore, as hospitals become overwhelmed, the possibility that some non-COVID patients are starting to be affected will increase, making the hospital to become more crowded and reducing access to medical facilities. India also experienced similar problems due to a delta variant surge. The high incidence of infection with this variant of COVID-19 was accompanied by a shortage of medical equipment and beds in hospitals in large cities^24^. Another defect in our health system was the poor quality of COVID-19 patients’ meals in hospitals, who were required high energetic and protein meal supply to promote healing times and decrease hospitalization periods. Luigia Brugliera declared that qualified nutritional protocols should be considered in the management of COVID-19 patients to improve their both short and long-term prognosisy^25^.

To the best of our knowledge, this study is the largest one to assess the risk of hospital admission during the alpha and delta surge among confirmed cases in Iran. However, several limitations were observed in this study. The general population vaccination in Iran started in May 2021, however, there was no information available regarding whether the patients admitted to the hospital were vaccinated. Analyses of hospitalization risks should also consider subgroup analyses for vaccinated and unvaccinated patients. In addition, disease severity levels were not recorded in the study, making it impossible to determine mortality rates based on severity levels. Furthermore, patients were classified into alpha and delta by the month in which they were admitted. The exact variant of COVID-19 should be identified and recorded in the data by special laboratory tests.

## Conclusion

In aggregate, the current study showed that the death proportion decreased from March to October 2021, while hospitalization and risk of COVID-19 death increased. Additionally, delta surge was associated with a higher risk of COVID-19 death compared to alpha surge. High replication efficiency in airway organoids and human epithelial systems, a high number of hospitalizations, a shortage of hospital beds, ICU spaces or medical supplies, poor nutritional status of hospitalized patients, burnout among health care providers, and lack of the intensivist physicians or experienced nurses in the ICU were factors that contributed to the high mortality rate in the delta surge in Iran.

## Supplementary Information

Supplementary Information.

## Data Availability

It is possible to obtain the data that supported the findings of this study from the Coronavirus Control Operations Headquarter in Tehran, however there are restrictions on access to these data, which were used under license for this study, and thus are not publicly available. Requests regarding the data may be made to the senior author.

## Abbreviations

SARS‐CoV‐2: Severe acute respiratory syndrome coronavirus 2
WHO: World Health Organization
IQR: Interquartile range
CI: Confidence interval
ICU: Intensive Care Unit
HIV: Human immunodeficiency virus
EF: Effect size
