# Supplementary material for: Mortality among hospitalized COVID-19 patients during surges of SARS-CoV-2 alpha (B.1.1.7) and delta (B.1.617.2) variants
Source: Sci Rep. 2022 Nov 7;12:18918. doi: 10.1038/s41598-022-23312-8 (PMC9640720; doi:10.1038/s41598-022-23312-8)
Supplement: Supplementary file 1 — Supplementary Information. [file 41598_2022_23312_MOESM1_ESM.docx]

**Supplementary**

Table S1. The proportion (95% CI) of patients’ characteristics at baseline, stratified by month of admission.

|  | March | April | May | June | July | August | September | October |
| --- | --- | --- | --- | --- | --- | --- | --- | --- |
| Total (No.) | 5743 | 37599 | 32483 | 25405 | 53128 | 70187 | 44007 | 2072 |
| Sex (No.,%) |  |  |  |  |  |  |  |  |
| Women | 48.1 (46.8, 49.4) | 48.2 (47.7, 48.8) | 48.6 (48.1, 49.2) | 48.8 (48.2, 49.4) | 49.1 (48.7, 49.6) | 50.8 (50.4, 51.1) | 51.8 (51.3, 52.2) | 52.7 (50.5, 54.8) |
| Men | 51.9 (50.6, 53.2) | 51.8 (51.2, 52.3) | 51.4 (50.8, 51.9) | 51.2 (50.6, 51.8) | 50.9 (50.4, 51.3) | 49.2 (48.9, 49.6) | 48.2 (47.8, 48.7) | 47.3 (45.2, 49.5) |
| Age (No. %) |  |  |  |  |  |  |  |  |
| <40 | 25.9 (24.8, 27.1) | 25.5 (25.1, 26.0) | 25.2 (24.7, 25.7) | 27.6 (27.1, 28.2) | 29.9 (29.5, 30.3) | 31.4 (31.0, 31.7) | 32.4 (32.0, 32.9) | 33.2 (31.1, 35.2) |
| 40-49 | 15.2 (14.3, 16.1) | 16.5 (16.1, 16.8) | 16.4 (16.0, 16.8) | 16.5 (16.1, 17.0) | 19.3 (19.0, 19.7) | 20.0 (19.7, 20.3) | 21.0 (20.6, 21.4) | 18.9 (17.3, 20.7) |
| 50-59 | 17.2 (16.2, 18.2) | 19.1 (18.7, 19.5) | 18.5 (18.0, 18.9) | 18.4 (18.0, 18.9) | 20.3 (19.9, 20.6) | 21.0 (20.7, 21.3) | 18.1 (17.8, 18.5) | 16.4 (14.8, 18.0) |
| 60-69 | 18.8 (17.8, 19.8) | 18.7 (18.3, 19.1) | 18.5 (18.0, 18.9) | 18.1 (17.7, 18.6) | 17.9 (17.5, 18.2) | 16.5 (16.3, 16.8) | 13.3 (13.0, 13.6) | 13.5 (12.1, 15.1) |
| 70-79 | 13.5 (12.7, 14.5) | 12.2 (11.9, 12.5) | 12.9 (12.6, 13.3) | 11.7 (11.3, 12.1) | 7.9 (7.6, 8.1) | 7.0 (6.9, 7.2) | 8.9 (8.6, 9.1) | 10.9 (9.6, 12.3) |
| 80-89 | 7.9 (7.2, 8.6) | 6.8 (6.6, 7.1) | 7.2 (7.0, 7.5) | 6.3 (6.0, 6.6) | 4.1 (3.9, 4.2) | 3.4 (3.3, 3.5) | 5.3 (5.1, 5.5) | 6.1 (5.2, 7.3) |
| >89 | 1.5 (1.2, 1.9) | 1.2 (1.1, 1.3) | 1.3 (1.2, 1.5) | 1.3 (1.2, 1.4) | 0.7 (0.6, 0.8) | 0.7 (0.6, 0.7) | 1.1 (1.0, 1.2) | 1.0 (0.6, 1.6) |
| Nationality (No.,%) |  |  |  |  |  |  |  |  |
| Iranian | 96.7 (96.2, 97.1) | 97.4 (97.2, 97.5) | 97.6 (97.4, 97.7) | 96.3 (96.0, 96.5) | 96.7 (96.5, 96.8) | 97.3 (97.1, 97.4) | 96.8 (96.6, 96.9) | 96.7 (95.8, 97.4) |
| Non-Iranian | 3.3 (2.9, 3.8) | 2.6 (2.5, 2.8) | 2.4 (2.3, 2.6) | 3.7 (3.5, 4.0) | 3.3 (3.2, 3.5) | 2.7 (2.6, 2.9) | 3.2 (3.1, 3.4) | 3.3 (2.6, 4.2) |
| Sign and Symptoms (No.%) |  |  |  |  |  |  |  |  |
| Vomit | 5.2 (4.6, 5.8) | 4.1 (3.9, 4.3) | 4.4 (4.2, 4.6) | 4.5 (4.3, 4.8) | 3.1 (2.9, 3.2) | 2.8 (2.6, 2.9) | 3.3 (3.2, 3.5) | 2.9 (2.3, 3.8) |
| Diarrhea | 4.6 (4.1, 5.2) | 3.9 (3.7, 4.1) | 3.7 (3.5, 3.9) | 4.0 (3.7, 4.2) | 2.6 (2.5, 2.8) | 2.3 (2.2, 2.4) | 2.3 (2.2, 2.5) | 1.6 (1.1, 2.3) |
| Anorexia | 10.2 (9.5, 11.1) | 9.5 (9.2, 9.8) | 9.6 (9.3, 9.9) | 9.7 (9.4, 10.1) | 9.0 (8.7, 9.2) | 10.2 (10.0, 10.4) | 9.5 (9.2, 9.8) | 9.3 (8.1, 10.7) |
| Paralysis | 0.2 (0.1, 0.3) | 0.1 (0.1, 0.1) | 0.1 (0.1, 0.2) | 0.1 (0.1, 0.2) | 0.1 (0.1, 0.1) | 0.1 (0.1, 0.1) | 0.1 (0.1, 0.1) | 0.0 (0.0, 0.2) |
| Fever | 38.2 (36.9, 39.5) | 36.0 (35.5, 36.5) | 35.2 (34.7, 35.7) | 39.0 (38.4, 39.6) | 40.1 (39.6, 40.5) | 39.7 (39.3, 40.1) | 38.9 (38.5, 39.4) | 34.5 (32.5, 36.6) |
| Seizure | 0.6 (0.4, 0.8) | 0.3 (0.2, 0.3) | 0.3 (0.3, 0.4) | 0.4 (0.3, 0.5) | 0.3 (0.2, 0.3) | 0.2 (0.2, 0.3) | 0.3 (0.2, 0.3) | 0.6 (0.3, 1.1) |
| Muscular pain | 37.4 (36.1, 38.7) | 36.2 (35.7, 36.7) | 35.8 (35.3, 36.3) | 34.6 (34.0, 35.2) | 37.8 (37.4, 38.2) | 39.1 (38.7, 39.4) | 40.2 (39.7, 40.6) | 39.0 (36.9, 41.2) |
| Chest pain | 3.4 (3.0, 4.0) | 3.8 (3.6, 4.0) | 3.1 (2.9, 3.3) | 2.8 (2.6, 3.0) | 2.2 (2.1, 2.3) | 2.4 (2.3, 2.5) | 2.5 (2.3, 2.6) | 2.0 (1.4, 2.7) |
| Abdominal pain | 2.5 (2.1, 2.9) | 3.1 (2.9, 3.3) | 2.5 (2.3, 2.7) | 2.5 (2.3, 2.7) | 2.2 (2.1, 2.3) | 2.4 (2.3, 2.5) | 2.1 (2.0, 2.2) | 1.4 (1.0, 2.1) |
| Dyspnea | 46.6 (45.3, 47.9) | 47.6 (47.1, 48.1) | 43.4 (42.8, 43.9) | 39.0 (38.4, 39.6) | 41.4 (41.0, 41.8) | 38.9 (38.6, 39.3) | 37.3 (36.8, 37.7) | 40.1 (38.0, 42.3) |
| Nausea | 8.4 (7.7, 9.1) | 7.0 (6.7, 7.3) | 6.6 (6.3, 6.9) | 7.2 (6.8, 7.5) | 6.5 (6.2, 6.7) | 7.1 (6.9, 7.3) | 6.8 (6.6, 7.1) | 6.6 (5.6, 7.8) |
| Headache | 9.0 (8.3, 9.8) | 10.2 (9.9, 10.5) | 10.7 (10.3, 11.0) | 9.7 (9.3, 10.1) | 11.0 (10.7, 11.2) | 11.2 (10.9, 11.4) | 11.4 (11.1, 11.7) | 9.9 (8.7, 11.3) |
| Cough | 52.2 (50.9, 53.5) | 54.2 (53.7, 54.7) | 54.7 (54.1, 55.2) | 53.5 (52.9, 54.1) | 58.9 (58.5, 59.4) | 63.3 (62.9, 63.6) | 62.0 (61.5, 62.4) | 57.8 (55.6, 59.9) |
| Vertigo | 3.2 (2.8, 3.7) | 3.6 (3.5, 3.8) | 3.6 (3.4, 3.8) | 3.2 (3.0, 3.4) | 3.1 (3.0, 3.3) | 3.2 (3.1, 3.4) | 3.2 (3.1, 3.4) | 3.9 (3.1, 4.9) |
| Skin lesion | 0.1 (0.0, 0.2) | 0.1 (0.1, 0.1) | 0.1 (0.1, 0.1) | 0.1 (0.1, 0.1) | 0.1 (0.1, 0.1) | 0.1 (0.1, 0.1) | 0.1 (0.1, 0.1) | 0.1 (0.0, 0.5) |
| loss of taste | 1.6 (1.3, 2.0) | 1.3 (1.2, 1.4) | 1.3 (1.1, 1.4) | 1.7 (1.5, 1.9) | 1.8 (1.7, 2.0) | 2.2 (2.1, 2.3) | 2.0 (1.9, 2.2) | 1.2 (0.8, 1.7) |
| Anosmia | 2.1 (1.7, 2.5) | 2.0 (1.9, 2.2) | 3.2 (3.0, 3.3) | 3.1 (2.9, 3.3) | 3.3 (3.1, 3.5) | 3.1 (3.0, 3.2) | 3.8 (3.6, 4.0) | 1.9 (1.4, 2.6) |
| Comorbidities (No.%) |  |  |  |  |  |  |  |  |
| Heart disease | 10.8 (10.0, 11.7) | 8.7 (8.5, 9.0) | 9.5 (9.2, 9.8) | 9.3 (8.9, 9.6) | 5.7 (5.5, 5.9) | 5.1 (5.0, 5.3) | 5.6 (5.3, 5.8) | 6.3 (5.3, 7.5) |
| HIV | 0.1 (0.0, 0.2) | 0.0 (0.0, 0.1) | 0.0 (0.0, 0.1) | 0.0 (0.0, 0.1) | 0.0 (0.0, 0.1) | 0.0 (0.0, 0.0) | 0.0 (0.0, 0.0) | 0.0 (0.0, 0.2) |
| Asthma | 1.0 (0.8, 1.3) | 1.0 (0.9, 1.1) | 0.9 (0.8, 1.1) | 0.9 (0.8, 1.0) | 0.6 (0.6, 0.7) | 0.5 (0.4, 0.5) | 0.5 (0.5, 0.6) | 0.4 (0.2, 0.9) |
| Neurological disease | 0.9 (0.7, 1.2) | 0.6 (0.5, 0.7) | 0.6 (0.6, 0.7) | 0.6 (0.5, 0.7) | 0.4 (0.3, 0.4) | 0.3 (0.3, 0.4) | 0.4 (0.4, 0.5) | 0.6 (0.3, 1.1) |
| Hypertension | 13.5 (12.6, 14.4) | 12.2 (11.9, 12.6) | 13.0 (12.6, 13.4) | 12.1 (11.7, 12.5) | 8.3 (8.1, 8.6) | 7.6 (7.4, 7.8) | 7.7 (7.5, 8.0) | 9.7 (8.5, 11.1) |
| Hematologic diseases | 0.5 (0.3, 0.7) | 0.3 (0.2, 0.3) | 0.3 (0.3, 0.4) | 0.4 (0.3, 0.5) | 0.3 (0.2, 0.3) | 0.3 (0.2, 0.3) | 0.3 (0.3, 0.4) | 0.5 (0.2, 0.9) |
| Liver disease | 0.5 (0.3, 0.7) | 0.3 (0.3, 0.4) | 0.4 (0.3, 0.5) | 0.4 (0.4, 0.5) | 0.3 (0.2, 0.3) | 0.2 (0.2, 0.3) | 0.3 (0.2, 0.3) | 0.1 (0.0, 0.5) |
| Kidney disease | 1.6 (1.3, 2.0) | 1.1 (1.0, 1.2) | 1.2 (1.1, 1.4) | 1.3 (1.1, 1.4) | 0.8 (0.7, 0.9) | 0.7 (0.6, 0.8) | 0.7 (0.6, 0.8) | 1.5 (1.0, 2.1) |
| Diabetes | 11.6 (10.8, 12.5) | 10.2 (9.9, 10.5) | 10.5 (10.2, 10.9) | 10.0 (9.6, 10.3) | 7.3 (7.1, 7.5) | 6.6 (6.4, 6.8) | 7.0 (6.7, 7.2) | 7.3 (6.2, 8.5) |
| Other chronic diseases | 5.5 (4.9, 6.1) | 4.2 (4.0, 4.4) | 5.0 (4.8, 5.3) | 5.0 (4.7, 5.3) | 3.4 (3.2, 3.5) | 3.0 (2.8, 3.1) | 3.5 (3.3, 3.6) | 4.2 (3.4, 5.2) |
| Cancer | 1.2 (1.0, 1.5) | 1.1 (1.0, 1.2) | 1.3 (1.2, 1.4) | 1.2 (1.1, 1.4) | 0.7 (0.6, 0.8) | 0.6 (0.5, 0.6) | 0.8 (0.7, 0.9) | 0.9 (0.5, 1.4) |
| Immunodeficiency | 0.3 (0.2, 0.5) | 0.2 (0.2, 0.2) | 0.3 (0.2, 0.3) | 0.2 (0.1, 0.2) | 0.1 (0.1, 0.2) | 0.1 (0.1, 0.2) | 0.2 (0.2, 0.3) | 0.3 (0.1, 0.7) |
| No. of comorbidities (No.%) |  |  |  |  |  |  |  |  |
| 0 | 69.0 (67.7, 70.1) | 73.2 (72.7, 73.6) | 71.7 (71.2, 72.2) | 73.0 (72.4, 73.5) | 81.1 (80.7, 81.4) | 83.0 (82.7, 83.3) | 82.0 (81.7, 82.4) | 79.0 (77.1, 80.7) |
| 1 | 18.5 (17.5, 19.5) | 16.6 (16.2, 17.0) | 17.1 (16.7, 17.5) | 16.2 (15.8, 16.7) | 12.0 (11.8, 12.3) | 10.8 (10.6, 11.0) | 11.0 (10.7, 11.3) | 13.0 (11.6, 14.6) |
| 2 | 9.2 (8.5, 10.0) | 7.6 (7.4, 7.9) | 8.1 (7.8, 8.4) | 7.8 (7.5, 8.2) | 5.2 (5.0, 5.4) | 4.6 (4.4, 4.7) | 5.2 (5.0, 5.5) | 5.6 (4.6, 6.6) |
| 3 | 2.8 (2.4, 3.3) | 2.2 (2.1, 2.4) | 2.7 (2.5, 2.9) | 2.6 (2.4, 2.8) | 1.4 (1.3, 1.5) | 1.4 (1.3, 1.5) | 1.4 (1.3, 1.6) | 2.1 (1.6, 2.9) |
| ≥4 | 0.6 (0.4, 0.8) | 0.3 (0.3, 0.4) | 0.5 (0.4, 0.5) | 0.4 (0.3, 0.5) | 0.3 (0.2, 0.3) | 0.2 (0.2, 0.3) | 0.3 (0.2, 0.3) | 0.3 (0.1, 0.7) |
| Pregnancy (No.%) |  |  |  |  |  |  |  |  |
| No | 99.1 (98.8, 99.3) | 99.3 (99.2, 99.4) | 99.4 (99.3, 99.5) | 99.4 (99.3, 99.5) | 99.5 (99.4, 99.5) | 99.5 (99.5, 99.6) | 99.4 (99.4, 99.5) | 99.5 (99.1, 99.8) |
| Yes | 0.9 (0.7, 1.2) | 0.7 (0.6, 0.8) | 0.6 (0.5, 0.7) | 0.6 (0.5, 0.7) | 0.5 (0.5, 0.6) | 0.5 (0.4, 0.5) | 0.6 (0.5, 0.6) | 0.5 (0.2, 0.9) |
| Smoking (No.%) |  |  |  |  |  |  |  |  |
| No | 98.2 (97.8, 98.5) | 98.7 (98.5, 98.8) | 98.7 (98.5, 98.8) | 98.4 (98.3, 98.6) | 98.8 (98.7, 98.9) | 99.1 (99.0, 99.1) | 99.0 (98.9, 99.1) | 99.0 (98.5, 99.4) |
| Yes | 1.8 (1.5, 2.2) | 1.3 (1.2, 1.5) | 1.3 (1.2, 1.5) | 1.6 (1.4, 1.7) | 1.2 (1.1, 1.3) | 0.9 (0.9, 1.0) | 1.0 (0.9, 1.1) | 1.0 (0.6, 1.5) |
| Drug abuse (No.%) |  |  |  |  |  |  |  |  |
| No | 99.1 (98.8, 99.3) | 99.4 (99.4, 99.5) | 99.4 (99.3, 99.5) | 99.2 (99.1, 99.3) | 99.6 (99.5, 99.7) | 99.7 (99.6, 99.7) | 99.6 (99.5, 99.6) | 99.6 (99.1, 99.8) |
| Yes | 0.9 (0.7, 1.2) | 0.6 (0.5, 0.6) | 0.6 (0.5, 0.7) | 0.8 (0.7, 0.9) | 0.4 (0.3, 0.5) | 0.3 (0.3, 0.4) | 0.4 (0.4, 0.5) | 0.4 (0.2, 0.9) |
| CT Scan (No.%) |  |  |  |  |  |  |  |  |
| Negative | 28.5 (27.4, 29.7) | 26.0 (25.5, 26.4) | 26.1 (25.6, 26.6) | 30.1 (29.5, 30.7) | 24.9 (24.5, 25.3) | 23.3 (23.0, 23.6) | 23.0 (22.6, 23.4) | 26.0 (24.1, 27.9) |
| Positive | 71.5 (70.3, 72.6) | 74.0 (73.6, 74.5) | 73.9 (73.4, 74.4) | 69.9 (69.3, 70.5) | 75.1 (74.7, 75.5) | 76.7 (76.4, 77.0) | 77.0 (76.6, 77.4) | 74.0 (72.1, 75.9) |
| Ward |  |  |  |  |  |  |  |  |
| Non-ICU treated | 78.0 (76.9, 79.1) | 85.4 (85.0, 85.7) | 86.3 (85.9, 86.6) | 84.9 (84.5, 85.3) | 88.6 (88.3, 88.8) | 91.4 (91.2, 91.6) | 89.5 (89.2, 89.8) | 89.1 (87.7, 90.4) |
| ICU treated | 22.0 (20.9, 23.1) | 14.6 (14.3, 15.0) | 13.7 (13.4, 14.1) | 15.1 (14.7, 15.5) | 11.4 (11.2, 11.7) | 8.6 (8.4, 8.8) | 10.5 (10.2, 10.8) | 10.9 (9.6, 12.3) |
| Death status |  |  |  |  |  |  |  |  |
| Survived | 90.1 (89.3, 90.8) | 90.4 (90.1, 90.7) | 92.5 (92.2, 92.8) | 94.0 (93.7, 94.3) | 92.7 (92.5, 92.9) | 93.6 (93.4, 93.8) | 95.2 (95.0, 95.4) | 99.3 (98.8, 99.6) |
| Deceased | 9.9 (9.2, 10.7) | 9.6 (9.3, 9.9) | 7.5 (7.2, 7.8) | 6.0 (5.7, 6.3) | 7.3 (7.1, 7.5) | 6.4 (6.2, 6.6) | 4.8 (4.6, 5.0) | 0.7 (0.4, 1.2) |

Table S2. The proportion (95% CI) of death status in different characteristics of patients stratified by Ward

|  | **Non-ICU treated** | | **EF (p-value)** | **ICU treated** | | **EF (p-value)** |
| --- | --- | --- | --- | --- | --- | --- |
|  | **Survived** | **Deceased** |  | **Survived** | **Deceased** |  |
| Total (No. %) | 96.8 (96.7, 96.8) | 3.2 (3.2, 3.3) |  | 66.0 (65.5, 66.5) | 34.0 (33.5, 34.5) |  |
| Sex (No,%) |  |  | 0.02 (<0.001) |  |  | 0.03 (<0.001) |
| Women | 50.5 (50.3, 50.7) | 44.0 (42.8, 45.1) |  | 46.9 (46.2, 47.6) | 43.7 (42.8, 44.7) |  |
| Men | 49.5 (49.3, 49.7) | 56.0 (54.9, 57.2) |  | 53.1 (52.4, 53.8) | 56.3 (55.3, 57.2) |  |
| Age |  |  | 0.21 (<0.001) |  |  | 0.23 (<0.001) |
| <40 | 31.9 (31.7, 32.1) | 7.0 (6.4, 7.6) |  | 20.4 (19.9, 20.9) | 6.6 (6.2, 7.1) |  |
| 40-49 | 20.0 (19.8, 20.1) | 7.2 (6.6, 7.8) |  | 13.7 (13.3, 14.2) | 8.9 (8.4, 9.5) |  |
| 50-59 | 19.9 (19.8, 20.1) | 15.1 (14.3, 15.9) |  | 17.7 (17.2, 18.2) | 15.7 (15.0, 16.4) |  |
| 60-69 | 16.0 (15.8, 16.1) | 24.6 (23.6, 25.6) |  | 21.1 (20.6, 21.7) | 25.2 (24.4, 26.0) |  |
| 70-79 | 8.0 (7.9, 8.1) | 21.6 (20.6, 22.5) |  | 15.4 (14.9, 15.9) | 21.9 (21.1, 22.7) |  |
| 80-89 | 3.7 (3.6, 3.8) | 19.6 (18.7, 20.5) |  | 9.7 (9.3, 10.1) | 17.2 (16.5, 18.0) |  |
| >89 | 0.6 (0.6, 0.6) | 5.0 (4.5, 5.5) |  | 2.0 (1.8, 2.2) | 4.5 (4.1, 4.9) |  |
| Nationality (No.,%) |  |  | -0.1 (<0.001) |  |  | 0.00 (0.651) |
| Iranian | 97.1 (97.0, 97.2) | 96.0 (95.5, 96.4) |  | 96.7 (96.4, 96.9) | 96.6 (96.2, 96.9) |  |
| Non-Iranian | 2.9 (2.8, 3.0) | 4.0 (3.6, 4.5) |  | 3.3 (3.1, 3.6) | 3.4 (3.1, 3.8) |  |
| Sign and Symptoms (No.%) |  |  |  |  |  |  |
| Vomit | 3.6 (3.5, 3.6) | 2.1 (1.8, 2.4) | 0.01 (<0.001) | 3.6 (3.4, 3.9) | 3.4 (3.1, 3.8) | 0.00 (0.390) |
| Diarrhea | 3.1 (3.0, 3.2) | 1.6 (1.3, 1.9) | 0.02 (<0.001) | 2.4 (2.2, 2.7) | 2.1 (1.9, 2.4) | 0.01 (0.082) |
| Anorexia | 9.6 (9.5, 9.7) | 7.5 (6.9, 8.1) | 0.01 (<0.001) | 9.2 (8.8, 9.6) | 11.9 (11.3, 12.6) | 0.04 (<0.001) |
| Paralysis | 0.1 (0.1, 0.1) | 0.2 (0.1, 0.3) | 0.01 (0.001) | 0.2 (0.2, 0.3) | 0.3 (0.2, 0.5) | 0.01 (0.077) |
| Fever | 39.3 (39.1, 39.5) | 35.1 (34.0, 36.1) | 0.02 (<0.001) | 33.3 (32.7, 33.9) | 33.8 (32.9, 34.7) | 0.00 (0.376) |
| Seizure | 0.3 (0.2, 0.3) | 0.1 (0.0, 0.2) | 0.01 (0.006) | 0.5 (0.4, 0.6) | 0.2 (0.2, 0.3) | 0.02 (<0.001) |
| Muscular pain | 38.6 (38.4, 38.8) | 32.0 (31.0, 33.1) | 0.02 (<0.001) | 32.3 (31.6, 32.9) | 34.2 (33.3, 35.1) | 0.02 (<0.001) |
| Chest pain | 2.3 (2.2, 2.4) | 2.1 (1.8, 2.5) | 0.00 (0.263) | 6.6 (6.2, 6.9) | 4.0 (3.7, 4.4) | 0.05 (<0.001) |
| Abdominal pain | 2.5 (2.4, 2.6) | 1.8 (1.5, 2.1) | 0.01 (<0.001) | 2.0 (1.8, 2.2) | 2.1 (1.9, 2.4) | 0.00 (0.470) |
| Dyspnea | 37.9 (37.7, 38.1) | 62.4 (61.3, 63.5) | 0.09 (<0.001) | 54.1 (53.4, 54.8) | 68.5 (67.6, 69.4) | 0.14 (<0.001) |
| Nausea | 7.1 (6.9, 7.2) | 3.8 (3.4, 4.3) | 0.02 (<0.001) | 6.3 (5.9, 6.6) | 6.6 (6.2, 7.1) | 0.01 (0.230) |
| Headache | 11.3 (11.2, 11.4) | 5.8 (5.3, 6.3) | 0.03 (<0.001) | 7.8 (7.4, 8.1) | 8.5 (8.0, 9.1) | 0.01 (0.019) |
| Cough | 60.4 (60.2, 60.6) | 47.3 (46.2, 48.4) | 0.05 (<0.001) | 47.9 (47.3, 48.6) | 51.5 (50.5, 52.4) | 0.03 (<0.001) |
| Vertigo | 3.3 (3.2, 3.4) | 2.1 (1.8, 2.4) | 0.01 (<0.001) | 3.7 (3.5, 4.0) | 3.8 (3.5, 4.2) | 0.00 (0.648) |
| Skin lesion | 0.1 (0.1, 0.1) | 0.1 (0.0, 0.2) | 0.00 (0.969) | 0.1 (0.1, 0.2) | 0.1 (0.1, 0.2) | 0.00 (0.788) |
| loss of taste | 1.8 (1.8, 1.9) | 0.5 (0.4, 0.7) | 0.02 (<0.001) | 2.0 (1.8, 2.2) | 1.5 (1.3, 1.8) | 0.02 (0.005) |
| Anosmia | 3.2 (3.1, 3.3) | 1.8 (1.5, 2.1) | 0.01 (<0.001) | 2.6 (2.4, 2.8) | 2.8 (2.5, 3.2) | 0.01 (0.196) |
| Comorbidities (No.%) |  |  |  |  |  |  |
| Heart disease | 5.3 (5.3, 5.4) | 14.0 (13.2, 14.8) | 0.07 (<0.001) | 15.2 (14.7, 15.7) | 17.4 (16.7, 18.1) | 0.03 (<0.001) |
| HIV | 0.0 (0.0, 0.0) | 0.1 (0.0, 0.2) | 0.00 (0.047) | 0.1 (0.0, 0.1) | 0.1 (0.1, 0.2) | 0.01 (0.221) |
| Asthma | 0.6 (0.6, 0.7) | 0.8 (0.7, 1.1) | 0.00 (0.030) | 1.1 (0.9, 1.2) | 1.0 (0.9, 1.3) | 0.00 (0.858) |
| Neurological disease | 0.4 (0.4, 0.4) | 0.8 (0.6, 1.0) | 0.01 (<0.001) | 0.9 (0.8, 1.0) | 1.3 (1.1, 1.5) | 0.02 (<0.001) |
| Hypertension | 8.1 (8.0, 8.2) | 15.3 (14.5, 16.1) | 0.05 (<0.001) | 18.1 (17.6, 18.6) | 22.0 (21.2, 22.8) | 0.05 (<0.001) |
| Hematologic diseases | 0.3 (0.3, 0.3) | 0.5 (0.3, 0.7) | 0.01 (<0.001) | 0.5 (0.4, 0.6) | 0.5 (0.4, 0.7) | 0.00 (0.900) |
| Liver disease | 0.3 (0.2, 0.3) | 0.7 (0.5, 0.9) | 0.01 (<0.001) | 0.5 (0.4, 0.6) | 0.6 (0.5, 0.8) | 0.01 (0.033) |
| Kidney disease | 0.7 (0.7, 0.7) | 2.3 (2.0, 2.7) | 0.03 (<0.001) | 1.6 (1.4, 1.8) | 3.4 (3.1, 3.8) | 0.06 (<0.001) |
| Diabetes | 7.0 (6.8, 7.1) | 15.4 (14.6, 16.2) | 0.06 (<0.001) | 14.3 (13.8, 14.8) | 17.7 (17.0, 18.5) | 0.05 (<0.001) |
| Other chronic diseases | 3.6 (3.5, 3.7) | 3.7 (3.3, 4.2) | 0.00 (0.675) | 4.6 (4.3, 4.9) | 5.9 (5.5, 6.4) | 0.03 (<0.001) |
| Cancer | 0.7 (0.6, 0.7) | 2.1 (1.8, 2.5) | 0.03 (<0.001) | 1.6 (1.5, 1.8) | 2.7 (2.4, 3.1) | 0.04 (<0.001) |
| Immunodeficiency | 0.2 (0.1, 0.2) | 0.2 (0.1, 0.3) | 0.00 (0.448) | 0.3 (0.2, 0.4) | 0.3 (0.2, 0.5) | 0.00 (0.575) |
| No. of comorbidities (No.%) |  |  | 0.08 (<0.001) |  |  | 0.07 (<0.001) |
| 0 | 81.4 (81.2, 81.5) | 65.9 (64.8, 67.0) |  | 62.9 (62.2, 63.5) | 56.5 (55.6, 57.4) |  |
| 1 | 12.0 (11.9, 12.2) | 17.8 (17.0, 18.7) |  | 21.0 (20.5, 21.6) | 22.3 (21.5, 23.1) |  |
| 2 | 5.0 (4.9, 5.1) | 11.7 (11.0, 12.5) |  | 11.4 (11.0, 11.9) | 14.5 (13.9, 15.2) |  |
| 3 | 1.4 (1.3, 1.4) | 3.7 (3.3, 4.2) |  | 4.0 (3.8, 4.3) | 5.3 (4.9, 5.8) |  |
| ≥4 | 0.2 (0.2, 0.2) | 0.8 (0.6, 1.1) |  | 0.6 (0.5, 0.8) | 1.4 (1.2, 1.6) |  |
| Pregnancy (No.%) |  |  | 0.01 (<0.001) |  |  | 0.04 (<0.001) |
| No | 99.4 (99.4, 99.4) | 99.9 (99.8, 100.0) |  | 99.4 (99.3, 99.5) | 99.9 (99.8, 100.0) |  |
| Yes | 0.6 (0.6, 0.6) | 0.1 (0.0, 0.2) |  | 0.6 (0.5, 0.7) | 0.1 (0.0, 0.2) |  |
| Smoking (No.%) |  |  | 0.00 (0.459) |  |  | 0.04 (<0.001) |
| No | 99.0 (99.0, 99.1) | 99.1 (98.9, 99.3) |  | 96.9 (96.6, 97.1) | 98.2 (97.9, 98.4) |  |
| Yes | 1.0 (0.9, 1.0) | 0.9 (0.7, 1.1) |  | 3.1 (2.9, 3.4) | 1.8 (1.6, 2.1) |  |
| Drug abuse (No.%) |  |  | 0.01 (<0.001) |  |  | 0.02 (<0.001) |
| No | 99.7 (99.7, 99.7) | 99.3 (99.1, 99.5) |  | 98.3 (98.1, 98.4) | 98.8 (98.6, 99.0) |  |
| Yes | 0.3 (0.3, 0.3) | 0.7 (0.5, 0.9) |  | 1.7 (1.6, 1.9) | 1.2 (1.0, 1.4) |  |
| CT Scan (No.%) |  |  | 0.02 (<0.001) |  |  | 0.11 (<0.001) |
| Negative | 25.2 (25.0, 25.4) | 20.8 (19.9, 21.7) |  | 28.3 (27.7, 28.9) | 18.2 (17.5, 18.9) |  |
| Positive | 74.8 (74.6, 75.0) | 79.2 (78.3, 80.1) |  | 71.7 (71.1, 72.3) | 81.8 (81.1, 82.5) |  |
| Hospitalization days. median (IQR) | 4 (2, 6) | 4 (2, 9) | 0.13 (<0.001) | 6 (4, 9) | 7 (3,12) | 0.21 (<0.001) |

Table S3. The hazard ratio of death due to COVID-19 among hospitalized patients. The Cox proportional model was adjusted for month of admission, sex, age, no. of comorbidities and nationality.

| **Variable** | **Levels** | **N** | **No. of events** | **HR (95% CI)** | **P-value** |
| --- | --- | --- | --- | --- | --- |
| Month | March (Ref) | 5724 | 569 | 1.00 |  |
|  | April | 33547 | 3595 | 1.15 (1.05 ,1.25) | 0.003 |
|  | May | 26850 | 2443 | 0.96 (0.88 ,1.05) | 0.385 |
|  | June | 20277 | 1522 | 0.87 (0.79 ,0.96) | 0.005 |
|  | July | 37028 | 3866 | 1.28 (1.17 ,1.40) | <0.001 |
|  | August | 39140 | 4486 | 1.40 (1.28 ,1.52) | <0.001 |
|  | September | 22756 | 2127 | 1.37 (1.25 ,1.50) | <0.001 |
|  | October | 294 | 15 | 4.63 (2.77 ,7.74) | <0.001 |
| Sex | Female (Ref) | 90985 | 8163 | 1.00 |  |
|  | Male | 94631 | 10460 | 1.17 (1.14 ,1.21) | <0.001 |
| Age | <40 (Ref) | 50632 | 1265 | 1.00 |  |
|  | 40-49 | 30432 | 1527 | 1.52 (1.41 ,1.63) | <0.001 |
|  | 50-59 | 34435 | 2875 | 2.09 (1.95 ,2.23) | <0.001 |
|  | 60-69 | 34239 | 4643 | 3.10 (2.91 ,3.30) | <0.001 |
|  | 70-79 | 21149 | 4049 | 4.35 (4.08 ,4.64) | <0.001 |
|  | 80-89 | 12313 | 3392 | 6.54 (6.13 ,6.99) | <0.001 |
|  | >89 | 2416 | 872 | 9.61 (8.81 ,10.49) | <0.001 |
| No. of comorbidities | 0 (Ref) | 135034 | 11248 | 1.00 |  |
|  | 1 | 30567 | 3804 | 1.15 (1.11 ,1.19) | <0.001 |
|  | 2 | 14673 | 2488 | 1.22 (1.17 ,1.27) | <0.001 |
|  | 3 | 4533 | 867 | 1.30 (1.21 ,1.39) | <0.001 |
|  | >=4 | 809 | 216 | 1.59 (1.39 ,1.82) | <0.001 |
| Nationality | Iranian (Ref) | 179403 | 17938 | 1.00 |  |
|  | Non-Iranian | 6213 | 685 | 1.68 (1.55 ,1.81) | <0.001 |

Abbreviation: hazard ratio (HR), confidence interval (CI).

Table S4. The unadjusted death proportion (95% confidence interval) due to COVID-19 among hospitalized patients per month.

| **Group** | **March** | **April** | **May** | **June** | **July** | **August** | **September** | **October** |
| --- | --- | --- | --- | --- | --- | --- | --- | --- |
| Total | 9.9 (9.2, 10.7) | 9.6 (9.3, 9.9) | 7.5 (7.2, 7.8) | 6.0 (5.7, 6.3) | 7.3 (7.1, 7.5) | 6.4 (6.2, 6.6) | 4.8 (4.6, 5.0) | 0.7 (0.4, 1.2) |
| Non-ICU treated | 4.0 (3.5, 4.6) | 4.9 (4.7, 5.1) | 3.1 (2.9, 3.4) | 2.1 (1.9, 2.3) | 3.6 (3.4, 3.8) | 3.2 (3.1, 3.4) | 2.2 (2.1, 2.4) | 0.2 (0.1, 0.6) |
| ICU treated | 30.8 (28.3, 33.4) | 36.8 (35.5, 38.1) | 35.0 (33.6, 36.4) | 28.0 (26.6, 29.5) | 35.9 (34.7, 37.1) | 39.8 (38.6, 41.1) | 26.8 (25.6, 28.1) | 4.9 (2.6, 8.8) |

Table S5. The mean and median survival time with 95% confidence interval by months

| **Month** | **Mean survival time (95 % CI)** | **Median survival time (95% CI)** | **P-value*** |
| --- | --- | --- | --- |
| March | 25.13 (23.48, 26.79) | 23.00 (20.76, 25.25) | <0.001 |
| April | 25.97 (25.13, 26.82) | 22.00 (21.02, 22.98) |  |
| May | 25.50 (24.48, 26.51) | 23.00 (21.84, 24.16) |  |
| June | 28.24 (26.50, 29.98) | 24.00 (22.44, 25.56) |  |
| July | 26.72 (25.65, 27.79) | 23.00 (22.11, 23.89) |  |
| August | 25.52 (24.51, 26.54) | 22.00 (21.27, 22.74) |  |
| September | 18.25 (17.76, 18.73) | 19.00 (18.37, 19.63) |  |
| October | 1.95 (1.93, 1.98) | --- |  |
| Overall | 25.98 (25.47, 26.49) | 22.00 (21.61, 22.39) |  |

* The log-rank test


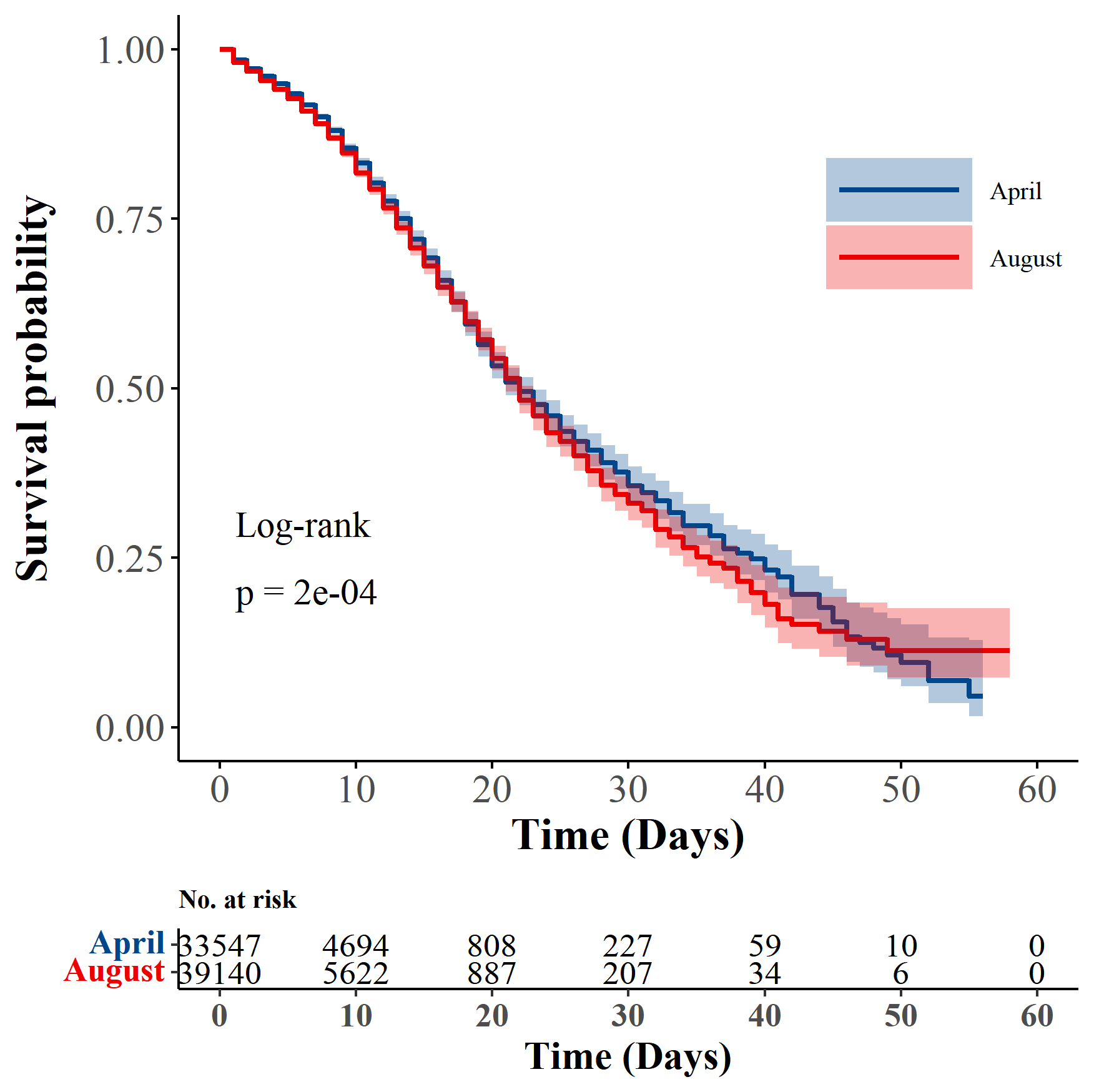


Figure S1. The Kaplan-Meier survival curve for both peaks occurred in April and August.

Table S6. The hazard ratio (95% CI) of death due to COVID-19 among hospitalized patients by wards.

| **Group** | **Month levels** | **N** | **No. of events** | **HR (95% CI)** | **P-value** |
| --- | --- | --- | --- | --- | --- |
| Total Patients |  |  |  |  |  |
|  | March (Ref) | 5724 | 569 | 1.00 | ---- |
|  | April | 33547 | 3595 | 1.15 (1.05, 1.25) | 0.003 |
|  | May | 26850 | 2443 | 0.96 (0.88, 1.05) | 0.385 |
|  | June | 20277 | 1522 | 0.87 (0.79, 0.96) | 0.005 |
|  | July | 37028 | 3866 | 1.28 (1.17, 1.40) | <0.001 |
|  | August | 39140 | 4486 | 1.40 (1.28, 1.52) | <0.001 |
|  | September | 22756 | 2127 | 1.37 (1.25, 1.50) | <0.001 |
|  | October | 294 | 15 | 4.63 (2.77, 7.74) | <0.001 |
| Non-ICU treated |  |  |  |  |  |
|  | March (Ref) | 4471 | 180 | 1.00 | ---- |
|  | April | 28090 | 1569 | 1.38 (1.18, 1.61) | <0.001 |
|  | May | 22418 | 881 | 0.95 (0.81, 1.12) | 0.572 |
|  | June | 16492 | 447 | 0.74 (0.62, 0.88) | 0.001 |
|  | July | 30991 | 1689 | 1.55 (1.32, 1.80) | <0.001 |
|  | August | 33131 | 2075 | 1.72 (1.47, 2.00) | <0.001 |
|  | September | 19030 | 884 | 1.45 (1.23, 1.70) | <0.001 |
|  | October | 269 | 4 | 2.24 (0.83, 6.04) | 0.111 |
| ICU-treated |  |  |  |  |  |
|  | March (Ref) | 1253 | 389 | 1.00 | ---- |
|  | April | 5457 | 2026 | 1.18 (1.06, 1.31) | 0.003 |
|  | May | 4432 | 1562 | 1.14 (1.02, 1.27) | 0.024 |
|  | June | 3785 | 1075 | 1.03 (0.92, 1.16) | 0.577 |
|  | July | 6037 | 2177 | 1.28 (1.15, 1.43) | <0.001 |
|  | August | 6009 | 2411 | 1.44 (1.29, 1.60) | <0.001 |
|  | September | 3726 | 1243 | 1.57 (1.40, 1.76) | <0.001 |
|  | October | 25 | 11 | 15.51 (8.50, 28.31) | <0.001 |

Note: All models were adjusted using variables including sex, age, no. of comorbidities, and nationality. Abbreviation: hazard ratio (HR), confidence interval (CI).


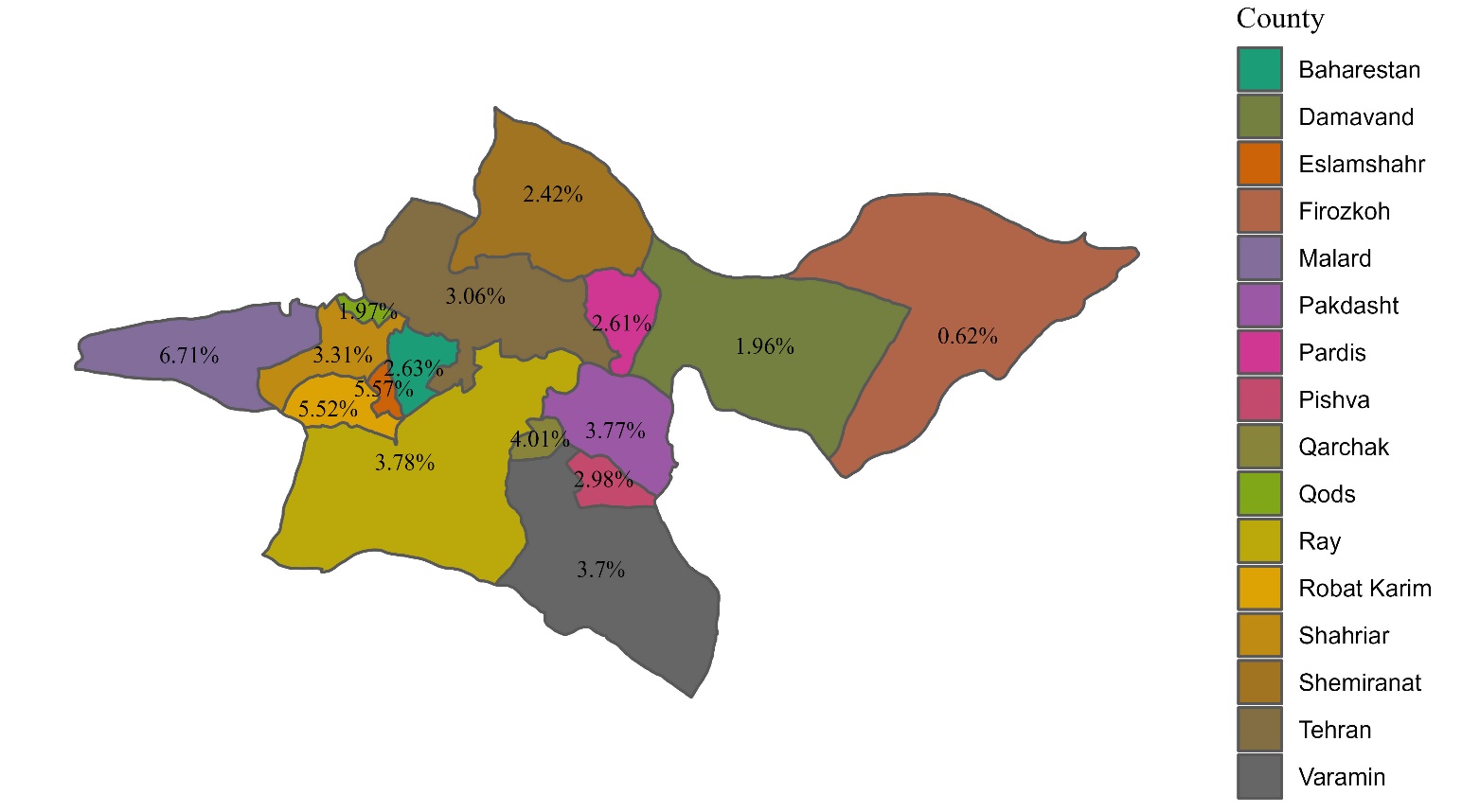


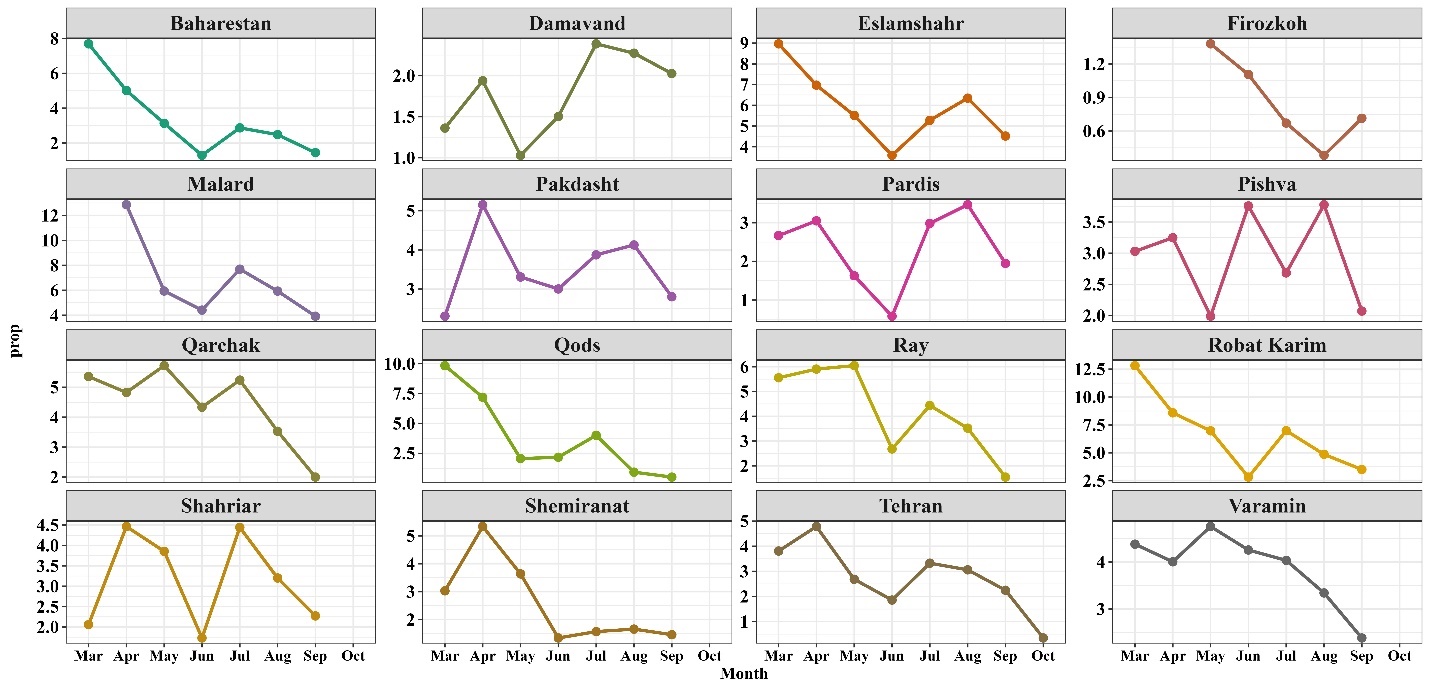


Figure S2. The overall death proportion (%) due to COVID-19 in non-ICU wards by counties of Tehran province (upper panel). The monthly death proportion due to COVID-19 with 95% confidence interval by counties of Tehran province (lower panel).


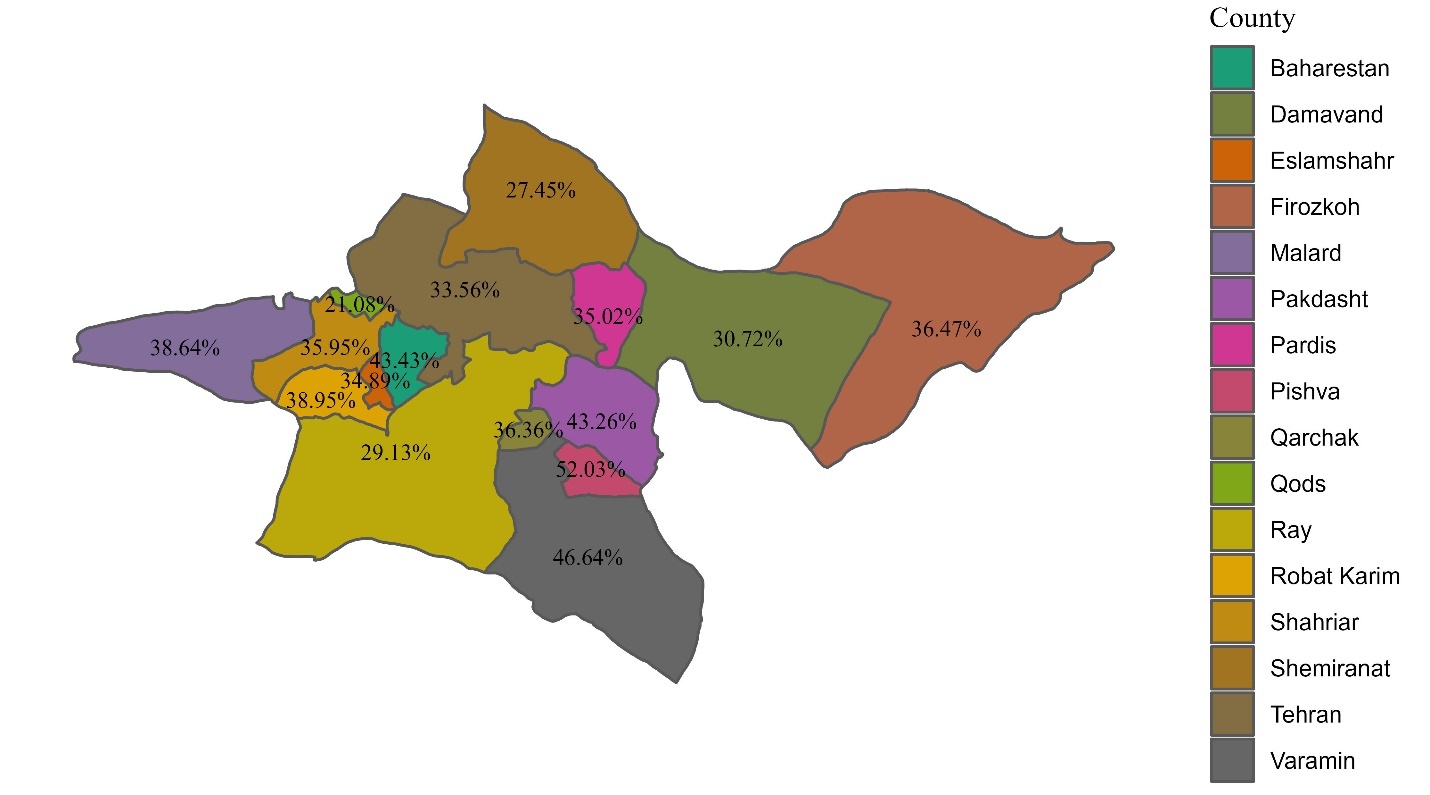


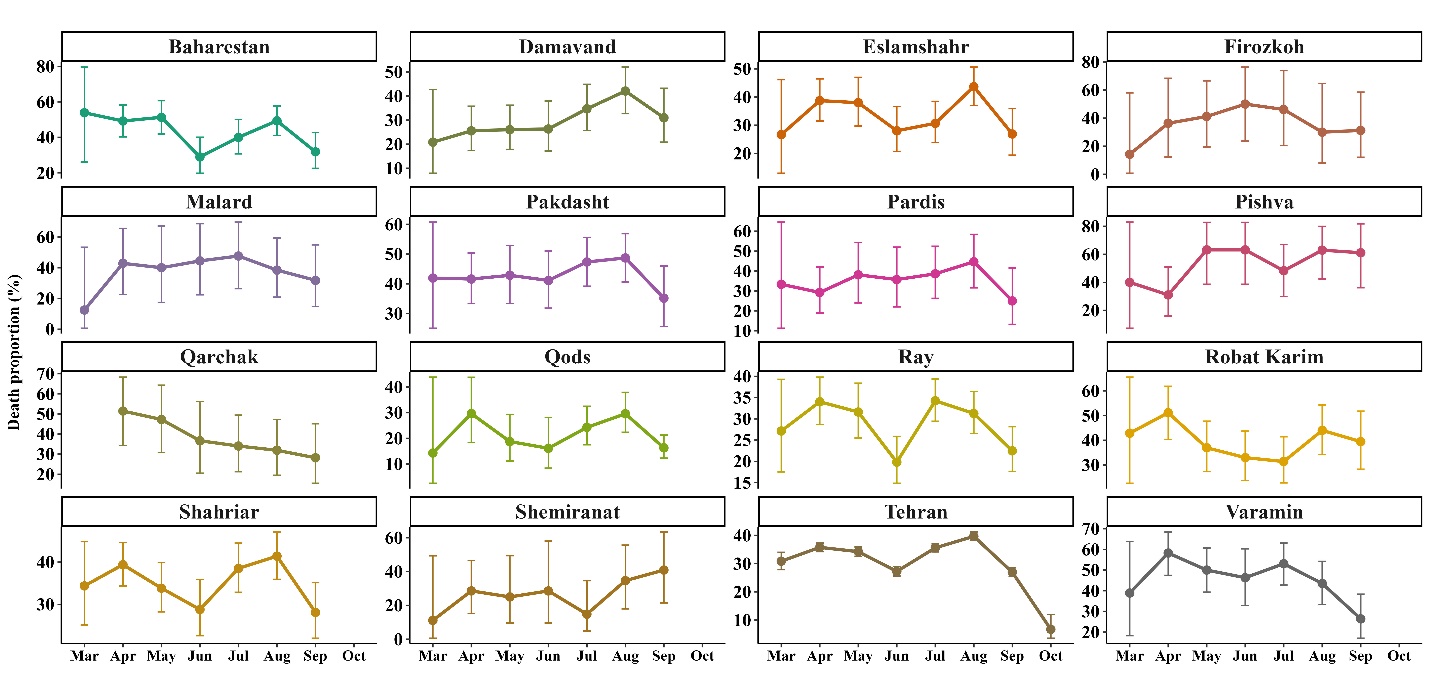


Figure S3. The overall death proportion (%) due to COVID-19 in ICU wards by counties of Tehran province (upper panel). The monthly death proportion due to COVID-19 with 95% confidence interval by counties of Tehran province (lower panel).


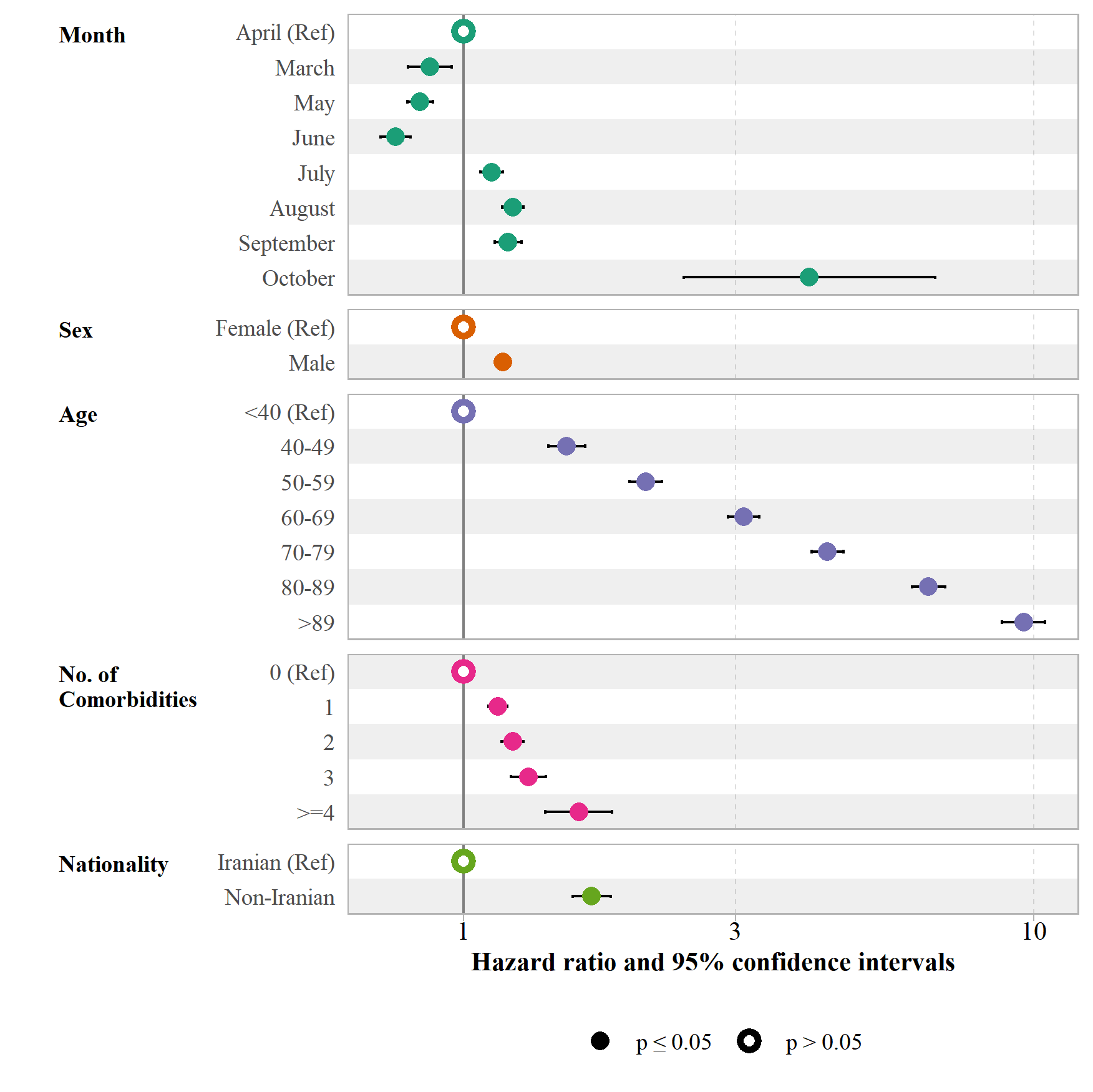

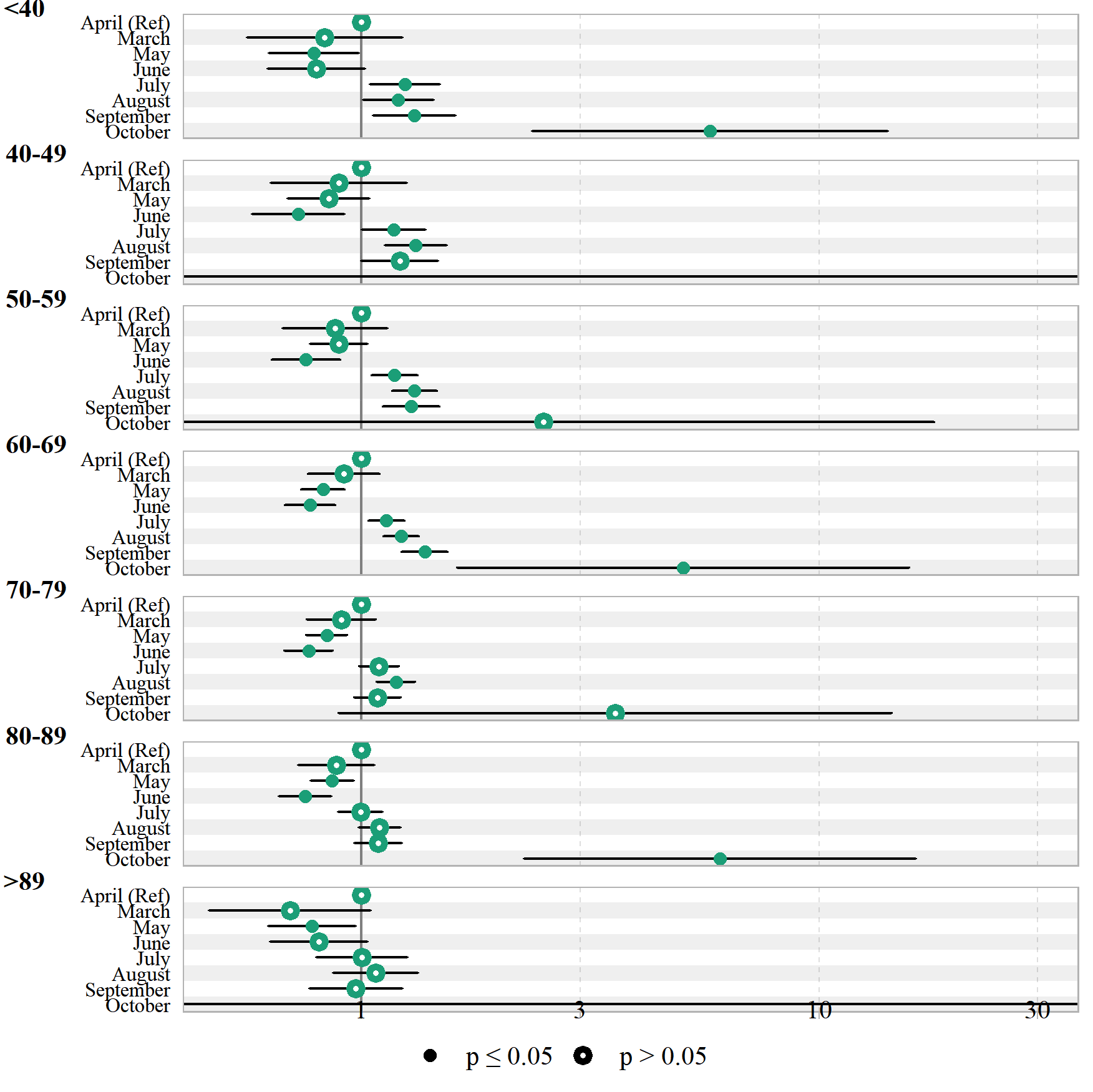


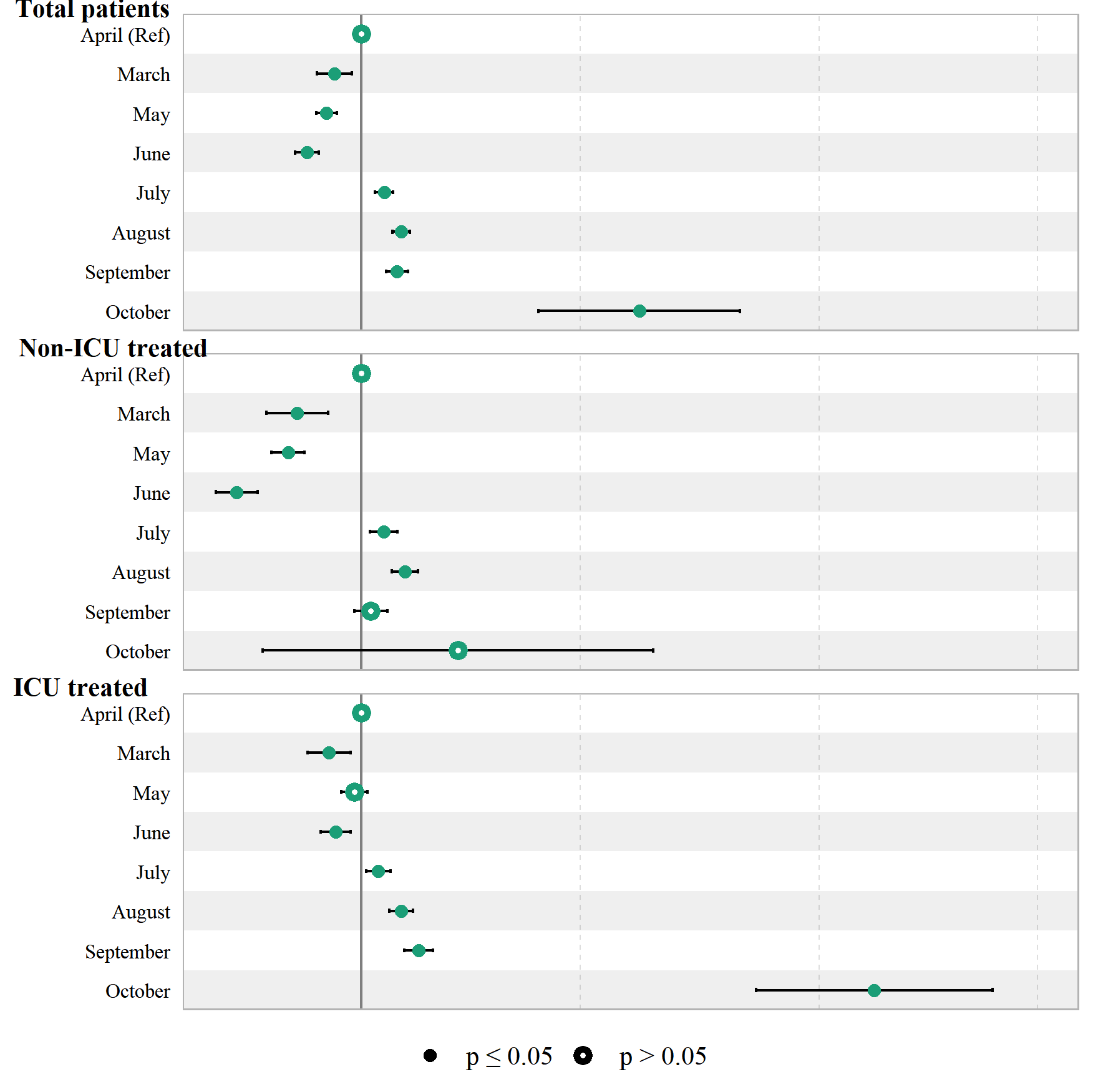


Figure S3. The subgroup HR and 95% CI of death among hospitalized COVID-19 patients by different group of age, sex, No. of comorbidities and nationality. [Note: April was the reference category in Month variable]

Table S7. The hazard ratio (95% CI) of death due to COVID-19 among hospitalized patients by wards.

| **Group** | **Month levels** | **N** | **No. of events** | **HR (95% CI)** | **P-value** |
| --- | --- | --- | --- | --- | --- |
| Total Patients |  |  |  |  |  |
|  | April (Ref) | 33547 | 3595 | 1.00 | ---- |
|  | March | 5724 | 569 | 0.87 (0.80, 0.95) | 0.003 |
|  | May | 26850 | 2443 | 0.84 (0.80, 0.88) | <0.001 |
|  | June | 20277 | 1522 | 0.76 (0.72, 0.81) | <0.001 |
|  | July | 37028 | 3866 | 1.12 (1.07, 1.17) | <0.001 |
|  | August | 39140 | 4486 | 1.22 (1.17, 1.27) | <0.001 |
|  | September | 22756 | 2127 | 1.20 (1.13, 1.26) | <0.001 |
|  | October | 294 | 15 | 4.04 (2.43, 6.72) | <0.001 |
| Non-ICU treated |  |  |  |  |  |
|  | April (Ref) | 28090 | 1569 | 1.00 | ---- |
|  | March | 4471 | 180 | 0.72 (0.62, 0.84) | <0.001 |
|  | May | 22418 | 881 | 0.69 (0.64, 0.75) | <0.001 |
|  | June | 16492 | 447 | 0.53 (0.48, 0.59) | <0.001 |
|  | July | 30991 | 1689 | 1.12 (1.04, 1.20) | 0.001 |
|  | August | 33131 | 2075 | 1.24 (1.16, 1.33) | <0.001 |
|  | September | 19030 | 884 | 1.05 (0.96, 1.14) | 0.271 |
|  | October | 269 | 4 | 1.62 (0.61, 4.33) | 0.334 |
| ICU-treated |  |  |  |  |  |
|  | April (Ref) | 5457 | 2026 | 1.00 | ---- |
|  | March | 1253 | 389 | 0.85 (0.76, 0.95) | 0.003 |
|  | May | 4432 | 1562 | 0.97 (0.90, 1.03) | 0.301 |
|  | June | 3785 | 1075 | 0.88 (0.82, 0.95) | <0.001 |
|  | July | 6037 | 2177 | 1.09 (1.02, 1.16) | 0.006 |
|  | August | 6009 | 2411 | 1.22 (1.15, 1.30) | <0.001 |
|  | September | 3726 | 1243 | 1.33 (1.24, 1.43) | <0.001 |
|  | October | 25 | 11 | 13.18 (7.27, 23.88) | <0.001 |

Note: All models were adjusted using variables including sex, age, no. of comorbidities, and nationality. Abbreviation: hazard ratio (HR), confidence interval (CI). [Note: April was the reference category in Month variable]

Table S8. The death proportion during study period by age groups

|  | **April** | **March**  **(First peak)** | **May** | **June** | **July** | **August**  **(Second peak)** | **September** | **October** |
| --- | --- | --- | --- | --- | --- | --- | --- | --- |
| <40 | 2.09  (1.82 2.40) | 1.95  (1.33 2.82) | 1.46  (1.21 1.75) | 1.35  (1.10 1.66) | 2.08  (1.86 2.31) | 1.45  (1.30 1.62) | 1.16  (1.00 1.36) | 0.73  (0.27 1.79) |
| 40-49 | 4.01  (3.54 4.53) | 4.36  (3.14 6.61) | 2.67  (2.26 3.15) | 2.40  (1.97 2.93) | 3.70  (3.34 4.79) | 3.14  (2.86 3.44) | 1.93  (1.66 2.24) |  |
| 50-59 | 6.80  (6.23 7.41) | 6.38  (4.97 8.13) | 4.89  (4.36 5.47) | 3.80  (3.27 4.49) | 6.56  (6.10 7.05) | 5.62  (5.25 6.19) | 4.01  (3.59 4.47) | 0.29  (0.02 1.89) |
| 60-69 | 12.47  (11.71 13.27) | 12.69  (10.79 14.85) | 8.60  (7.91 9.35) | 7.40  (6.66 8.29) | 11.32  (10.69 11.98) | 10.62  (10.06 11.26) | 7.92  (7.25 8.65) | 1.07  (0.28 3.36) |
| 70-79 | 19.04  (17.91 27.21) | 19.54  (16.84 22.54) | 15.35  (14.28 16.48) | 12.77  (11.59 14.73) | 17.19  (16.07 18.38) | 17.00  (15.97 18.39) | 11.25  (10.28 12.29) | 0.88  (0.15 3.53) |
| 80-89 | 28.77  (27.03 34.57) | 27.21  (23.21 31.61) | 24.56  (22.85 26.37) | 20.36  (18.43 22.44) | 24.38  (22.59 26.25) | 27.51  (25.73 29.35) | 18.95  (17.38 22.62) | 3.15  (1.01 8.36) |
| >89 | 37.86  (33.39 42.55) | 31.40  (22.05 42.42) | 34.40  (29.98 39.18) | 31.10  (26.19 36.46) | 34.85  (30.07 39.96) | 35.49  (31.24 39.98) | 26.39  (22.49 36.69) |  |


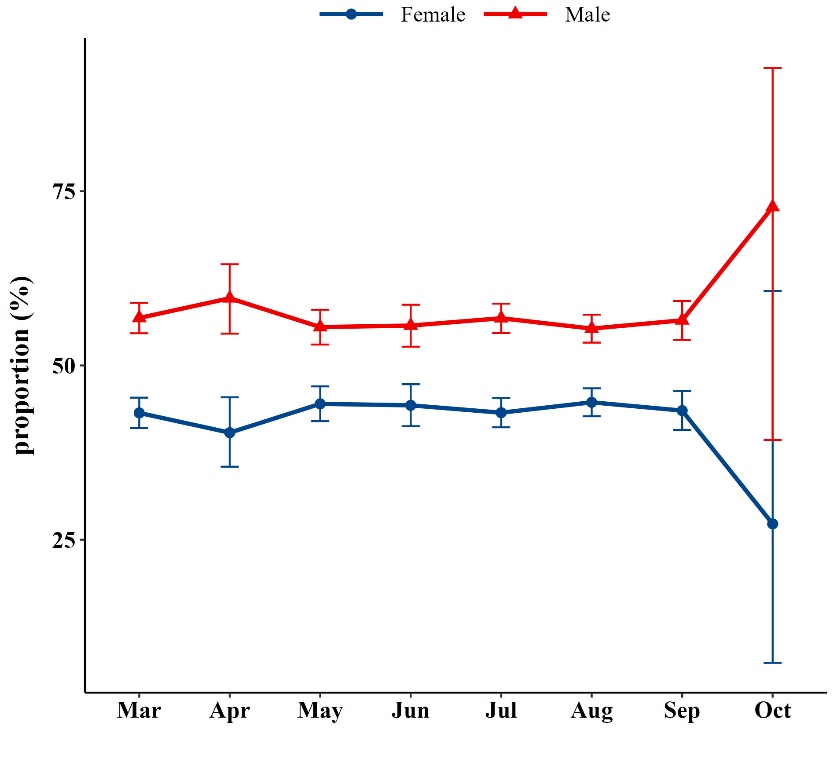

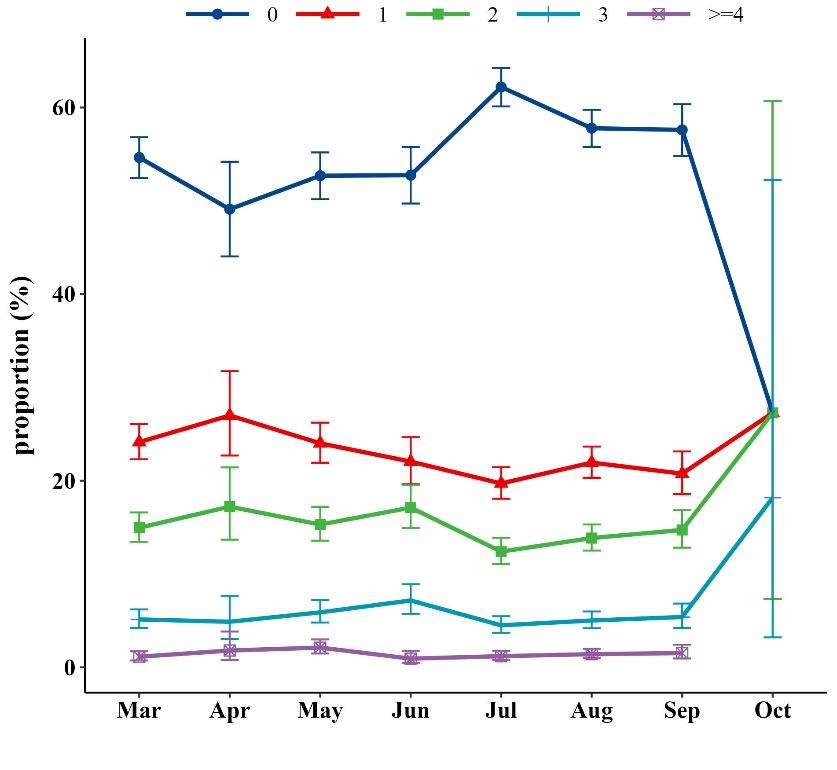


| 1. Sex | 1. Category of comorbidities |
| --- | --- |


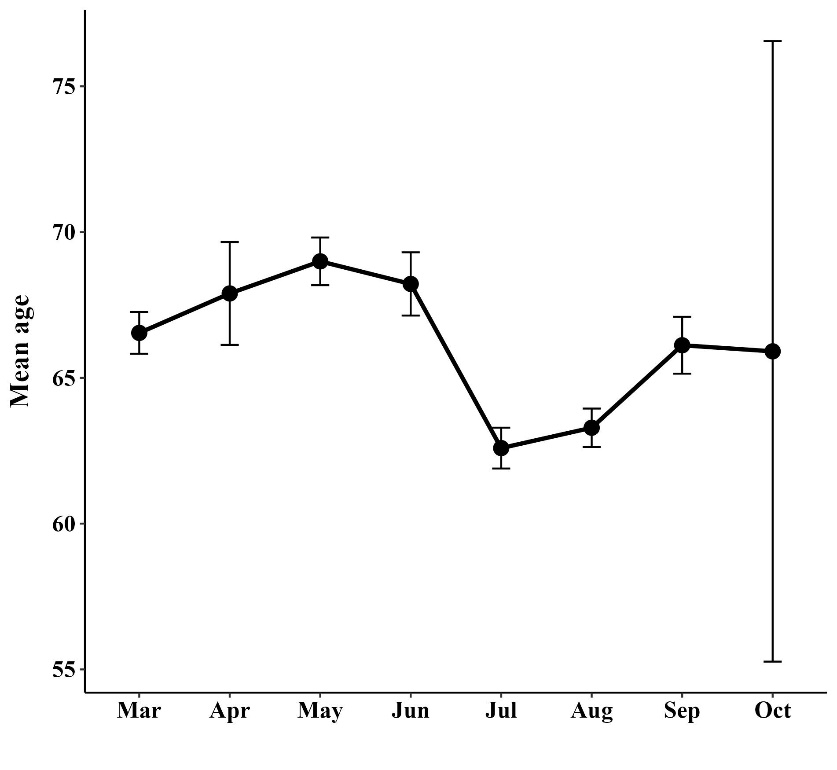

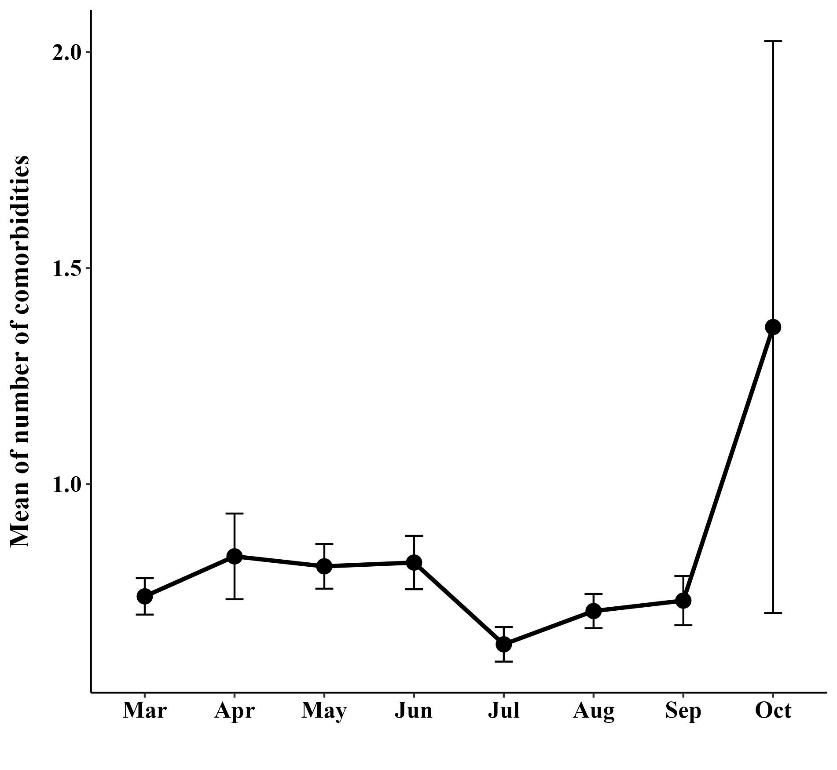


| 1. Mean age | 1. Mean number of comorbidities |
| --- | --- |

Figure S4. A) the proportion of men and women among ICU-treated by month, B) the proportion of comorbidities among ICU-treated, C) the mean age of patients in ICU by month, D) the mean number of comorbidities among ICU-treated by month

Table S9. The subgroup HR and 95% CI of death among hospitalized COVID-19 patients by different group of age

| **Age group** | **Month** | **HR (95% CI)** | **P-value** |
| --- | --- | --- | --- |
| <40 | March (Ref) | ---- | ---- |
|  | April | 1.20 (0.81, 1.78) | 0.353 |
|  | May | 0.95 (0.63, 1.42) | 0.789 |
|  | June | 0.96 (0.63, 1.45) | 0.842 |
|  | July | 1.50 (1.02, 2.19) | 0.038 |
|  | August | 1.45 (0.99, 2.11) | 0.058 |
|  | September | 1.57 (1.05, 2.33) | 0.026 |
|  | October | 6.85 (2.64, 17.80) | <0.001 |
| 40-49 | March (Ref) | ---- | ---- |
|  | April | 1.12 (0.80, 1.58) | 0.512 |
|  | May | 0.95 (0.66, 1.36) | 0.784 |
|  | June | 0.82 (0.56, 1.19) | 0.289 |
|  | July | 1.32 (0.94, 1.84) | 0.106 |
|  | August | 1.47 (1.06, 2.05) | 0.023 |
|  | September | 1.36 (0.95, 1.93) | 0.089 |
|  | October | 0.00 (0.00, 0.00) | 0.881 |
| 50-59 | March (Ref) | ---- | ---- |
|  | April | 1.14 (0.88, 1.49) | 0.320 |
|  | May | 1.02 (0.78, 1.34) | 0.891 |
|  | June | 0.87 (0.65, 1.15) | 0.321 |
|  | July | 1.35 (1.04, 1.74) | 0.024 |
|  | August | 1.49 (1.15, 1.92) | 0.002 |
|  | September | 1.46 (1.12, 1.92) | 0.006 |
|  | October | 2.82 (0.39, 20.38) | 0.304 |
| 60-69 | March (Ref) | ---- | ---- |
|  | April | 1.09 (0.91, 1.31) | 0.334 |
|  | May | 0.90 (0.75, 1.09) | 0.281 |
|  | June | 0.85 (0.69, 1.03) | 0.099 |
|  | July | 1.24 (1.03, 1.48) | 0.020 |
|  | August | 1.33 (1.12, 1.59) | 0.002 |
|  | September | 1.49 (1.23, 1.81) | <0.001 |
|  | October | 5.32 (1.69, 16.71) | 0.004 |
| 70-79 | March (Ref) | ---- | ---- |
|  | April | 1.11 (0.93, 1.32) | 0.249 |
|  | May | 0.93 (0.78, 1.11) | 0.422 |
|  | June | 0.85 (0.71, 1.03) | 0.095 |
|  | July | 1.21 (1.01, 1.44) | 0.037 |
|  | August | 1.31 (1.10, 1.56) | 0.002 |
|  | September | 1.20 (0.99, 1.44) | 0.059 |
|  | October | 3.80 (0.94, 15.37) | 0.061 |
| 80-89 | March (Ref) | ---- | ---- |
|  | April | 1.13 (0.94, 1.37) | 0.203 |
|  | May | 0.98 (0.81, 1.19) | 0.835 |
|  | June | 0.86 (0.70, 1.05) | 0.143 |
|  | July | 1.13 (0.93, 1.37) | 0.235 |
|  | August | 1.24 (1.02, 1.50) | 0.030 |
|  | September | 1.23 (1.00, 1.50) | 0.045 |
|  | October | 6.13 (2.26, 16.65) | <0.001 |
| >89 | March (Ref) | ---- | ---- |
|  | April | 1.42 (0.95, 2.13) | 0.091 |
|  | May | 1.12 (0.74, 1.68) | 0.602 |
|  | June | 1.15 (0.75, 1.76) | 0.511 |
|  | July | 1.42 (0.94, 2.15) | 0.096 |
|  | August | 1.53 (1.02, 2.29) | 0.042 |
|  | September | 1.38 (0.91, 2.10) | 0.128 |
|  | October | 0.00 (0.00, 0.00) | 0.928 |

Table S10. The subgroup HR and 95% CI of death among hospitalized COVID-19 patients by different group of sex

| **Age group** | **Month** | **HR (95% CI)** | **P-value** |
| --- | --- | --- | --- |
| Female | March (Ref) | ---- | ---- |
|  | April | 1.10 (0.96, 1.26) | 0.175 |
|  | May | 0.98 (0.85, 1.13) | 0.767 |
|  | June | 0.87 (0.75, 1.01) | 0.068 |
|  | July | 1.07 (0.93, 1.22) | 0.339 |
|  | August | 1.19 (1.04, 1.36) | 0.011 |
|  | September | 1.22 (1.06, 1.40) | 0.006 |
|  | October | 1.81 (0.68, 4.88) | 0.238 |
| Male | March (Ref) | ---- | ---- |
|  | April | 1.09 (0.97, 1.22) | 0.155 |
|  | May | 0.98 (0.87, 1.11) | 0.748 |
|  | June | 0.84 (0.74, 0.95) | 0.007 |
|  | July | 1.09 (0.97, 1.22) | 0.146 |
|  | August | 1.19 (1.06, 1.33) | 0.003 |
|  | September | 1.28 (1.14, 1.45) | <0.001 |
|  | October | 4.25 (2.33, 7.76) | <0.001 |

Table S11. The subgroup HR and 95% CI of death among hospitalized COVID-19 patients by different group of number of comorbidities

| **No. of Comorbidity** | **Month** | **HR (95% CI)** | **P-value** |
| --- | --- | --- | --- |
| 0 | March (Ref) | ---- | ---- |
|  | April | 1.19 (1.05, 1.34) | 0.006 |
|  | May | 1.04 (0.92, 1.18) | 0.538 |
|  | June | 0.91 (0.80, 1.04) | 0.158 |
|  | July | 1.20 (1.07, 1.36) | 0.003 |
|  | August | 1.28 (1.14, 1.45) | <0.001 |
|  | September | 1.35 (1.18, 1.53) | <0.001 |
|  | October | 2.05 (0.91, 4.61) | 0.082 |
| 1 | March (Ref) | ---- | ---- |
|  | April | 0.99 (0.83, 1.18) | 0.891 |
|  | May | 0.85 (0.71, 1.03) | 0.095 |
|  | June | 0.72 (0.59, 0.88) | 0.001 |
|  | July | 0.99 (0.83, 1.18) | 0.901 |
|  | August | 1.15 (0.96, 1.37) | 0.121 |
|  | September | 1.25 (1.03, 1.51) | 0.023 |
|  | October | 4.54 (1.68, 12.30) | 0.003 |
| 2 | March (Ref) | ---- | ---- |
|  | April | 1.05 (0.85, 1.31) | 0.636 |
|  | May | 0.96 (0.77, 1.20) | 0.712 |
|  | June | 0.86 (0.68, 1.08) | 0.199 |
|  | July | 1.08 (0.87, 1.34) | 0.499 |
|  | August | 1.20 (0.97, 1.49) | 0.093 |
|  | September | 1.18 (0.93, 1.48) | 0.172 |
|  | October | 7.13 (2.26, 22.57) | <0.001 |
| 3 | March (Ref) | ---- | ---- |
|  | April | 1.17 (0.78, 1.75) | 0.441 |
|  | May | 0.98 (0.65, 1.47) | 0.915 |
|  | June | 1.02 (0.67, 1.55) | 0.941 |
|  | July | 1.27 (0.85, 1.90) | 0.251 |
|  | August | 1.40 (0.94, 2.08) | 0.096 |
|  | September | 1.49 (0.98, 2.27) | 0.063 |
|  | October | 24.73 (5.84, 104.66) | <0.001 |
| >=4 | March (Ref) | ---- | ---- |
|  | April | 0.98 (0.46, 2.08) | 0.963 |
|  | May | 1.52 (0.74, 3.14) | 0.255 |
|  | June | 0.95 (0.41, 2.19) | 0.910 |
|  | July | 1.51 (0.73, 3.15) | 0.270 |
|  | August | 1.93 (0.95, 3.95) | 0.071 |
|  | September | 1.78 (0.83, 3.82) | 0.139 |
|  | October |  | 0.968 |

Table S12. The subgroup HR and 95% CI of death among hospitalized COVID-19 patients by different group of number of Nationality

| **Nationality** | **Month** | **HR (95% CI)** | **P-value** |
| --- | --- | --- | --- |
| Non-Iranian | March (Ref) | ---- | ---- |
|  | April | 0.91 (0.53, 1.57) | 0.738 |
|  | May | 0.85 (0.49, 1.49) | 0.575 |
|  | June | 0.85 (0.49, 1.48) | 0.565 |
|  | July | 1.18 (0.70, 2.00) | 0.527 |
|  | August | 1.06 (0.62, 1.81) | 0.820 |
|  | September | 1.28 (0.74, 2.21) | 0.373 |
|  | October |  | 0.926 |
| Iranian | March (Ref) | ---- | ---- |
|  | April | 1.10 (1.01, 1.20) | 0.038 |
|  | May | 0.98 (0.90, 1.08) | 0.722 |
|  | June | 0.85 (0.77, 0.94) | <0.001 |
|  | July | 1.07 (0.98, 1.17) | 0.130 |
|  | August | 1.19 (1.09, 1.30) | <0.001 |
|  | September | 1.24 (1.13, 1.37) | <0.001 |
|  | October | 3.36 (2.01, 5.61) | <0.001 |
